# Supplementary material for: GLDADec: marker-gene guided LDA modeling for bulk gene expression deconvolution
Source: Brief Bioinform. 2024 Jul 10;25(4):bbae315. doi: 10.1093/bib/bbae315 (PMC11233176; doi:10.1093/bib/bbae315)
Supplement: BIB_Supple_final_v2_bbae315 [file bib_supple_final_v2_bbae315.docx]

**Supplementary figures**

**GLDADec: marker-gene guided LDA modelling for bulk gene expression deconvolution**

*Iori Azuma^*, 1^, Tadahaya Mizuno^*, †, 2^, and Hiroyuki Kusuhara ^1^*

^1^Graduate School of Pharmaceutical Sciences, the University of Tokyo, Bunkyo, Tokyo, 113-0033, Japan

^2^Graduate School of Pharmaceutical Sciences, the University of Tokyo, Bunkyo, Tokyo, 113-0033, Japan, Electronic address: tadahaya@gmail.com

†Corresponding author: Tadahaya Mizuno, tadahaya@gmail.com

Graduate School of Pharmaceutical Sciences, the University of Tokyo, Bunkyo-ku, Tokyo, 113-0033, Japan

Tel: +81-3-5841-4771

*These authors contributed equally.

CONFLICT OF INTEREST

The authors declared no competing interests for this work.

FUNDING

This work was supported by JSPS KAKENHI Grant-in-Aid for Scientific Research (C) (grant number 21K06663) from the Japan Society for the Promotion of Science, Takeda Science Foundation, and Mochida Memorial Foundation for Medical and Pharmaceutical Research.

# **Figure S1**

**
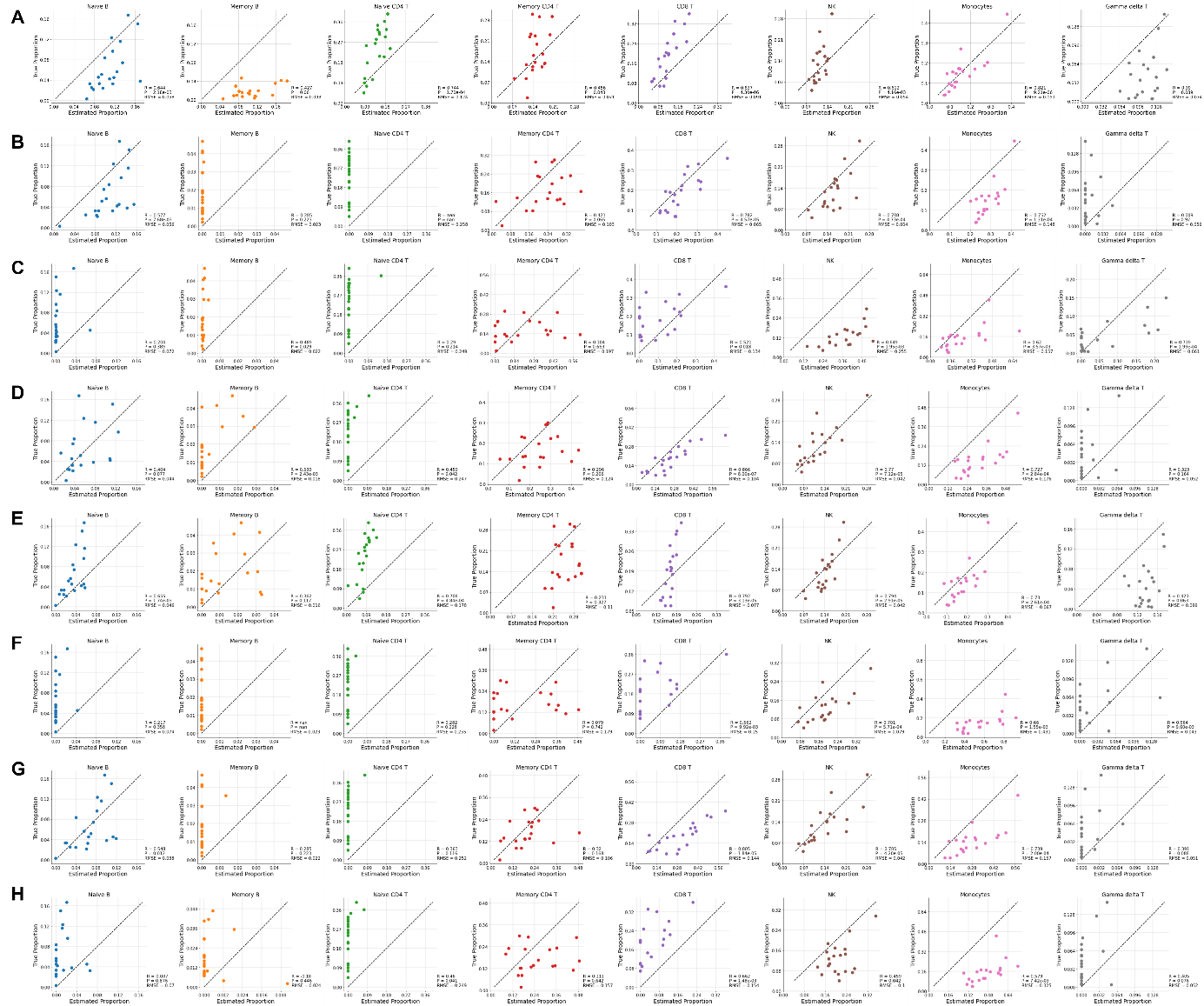
**

**Figure S1.** Scatterplots showing the correlation between estimated proportion and ground-truth proportion in the benchmark dataset GSE65133. Each row represents deconvolution method, (A) GLDADec, (B) FARDEEP, (C) EPIC, (D) CIBERSORT, (E) DCQ, (F) NNLS, (G) RLR, and (H) ElasticNet, respectively.

# **Figure S2**

**
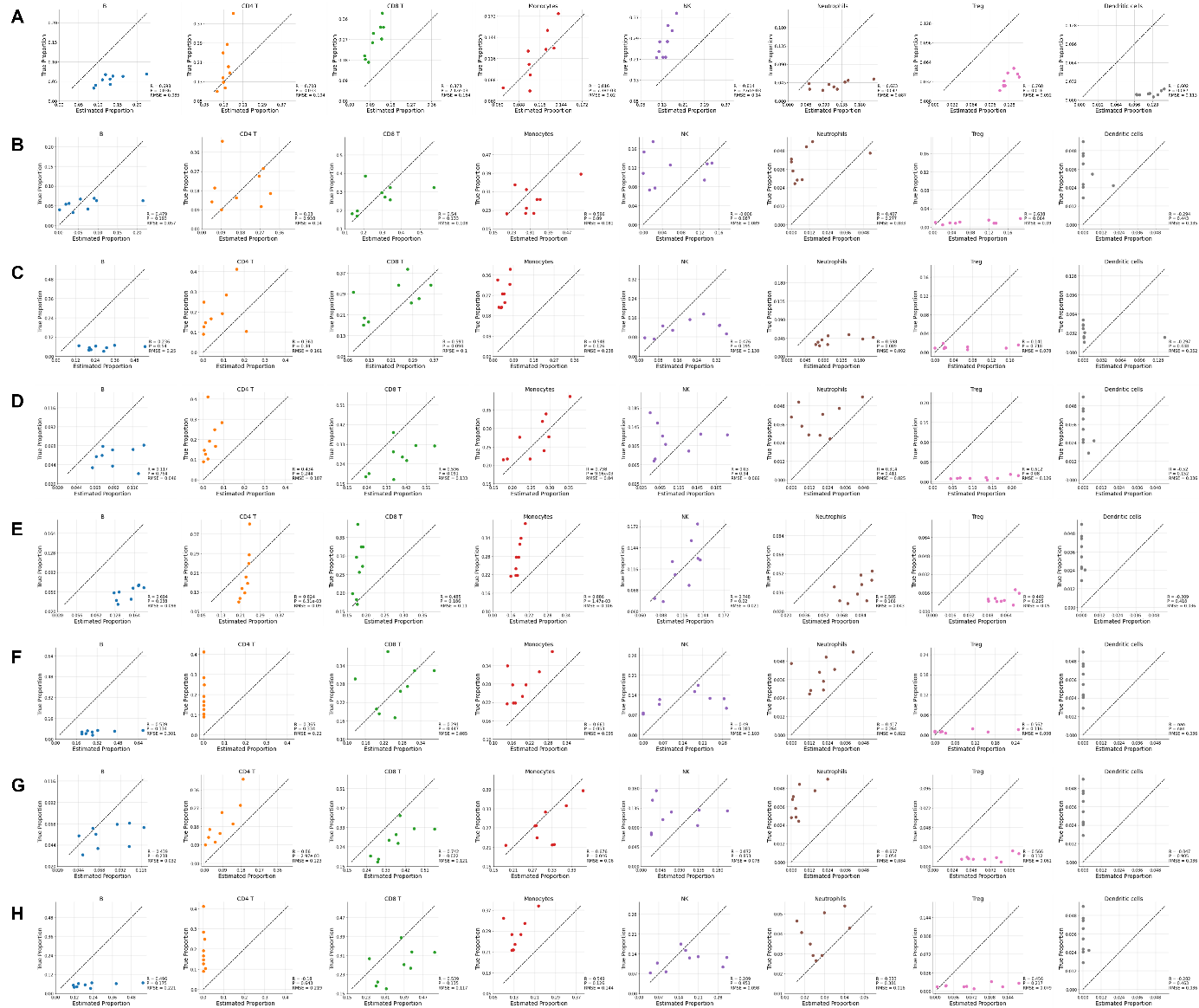
**

**Figure S2.** Scatterplots showing the correlation between estimated proportion and ground-truth proportion in the benchmark dataset GSE107572. Each row represents deconvolution method, (A) GLDADec, (B) FARDEEP, (C) EPIC, (D) CIBERSORT, (E) DCQ, (F) NNLS, (G) RLR, and (H) ElasticNet, respectively.

# **Figure S3**

**
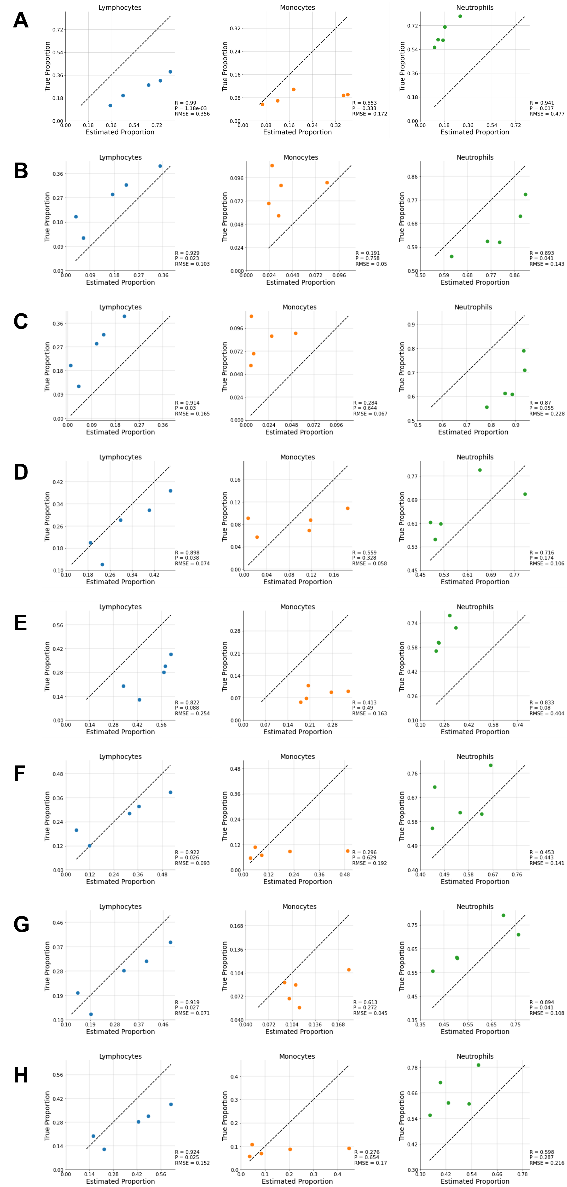
**

**Figure S3.** Scatterplots showing the correlation between estimated proportion and ground-truth proportion in the benchmark dataset GSE60424. Each row represents deconvolution method, (A) GLDADec, (B) FARDEEP, (C) EPIC, (D) CIBERSORT, (E) DCQ, (F) NNLS, (G) RLR, and (H) ElasticNet, respectively.

# **Figure S4**

**
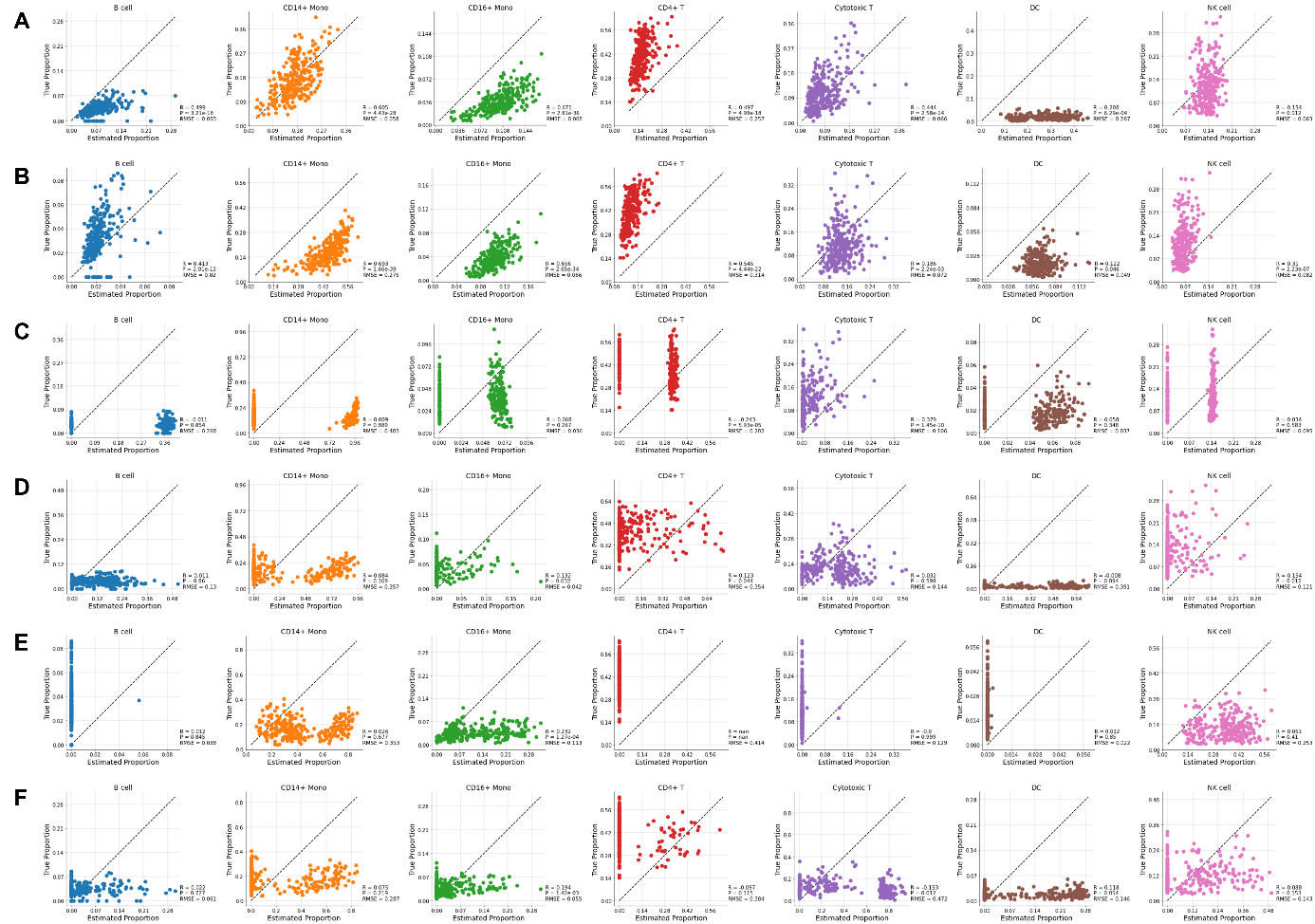
**

**Figure S4.** Scatterplots showing the correlation between estimated proportion and ground-truth proportion in the benchmark dataset SDY67. Each row represents deconvolution method, (A) GLDADec, (B) GTM-decon, (C) BayesPrism, (D) CIBERSORTx, (E) MuSiC, and (F) BSEQ-sc, respectively.

# **Figure S5**

**
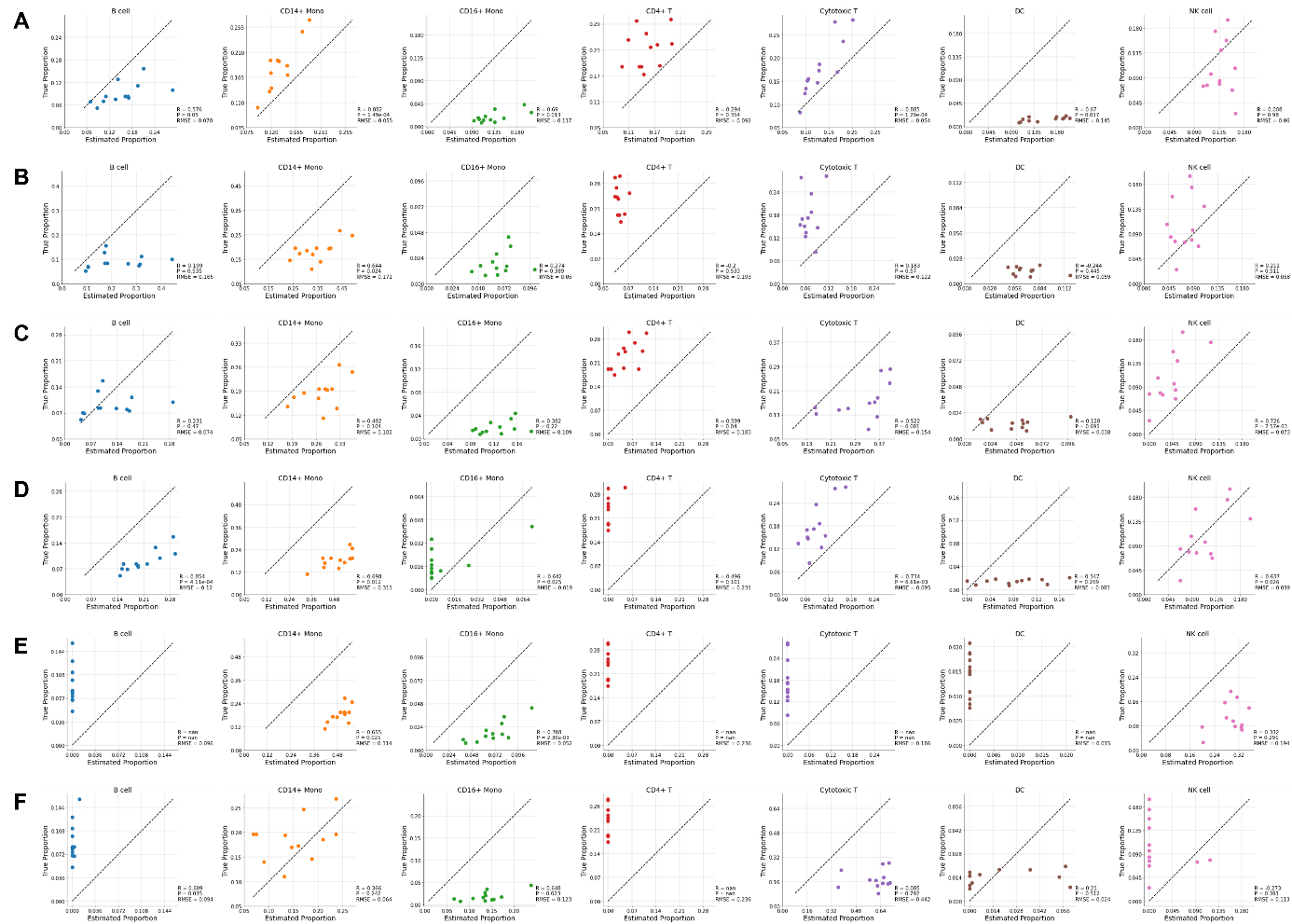
**

**Figure S5.** Scatterplots showing the correlation between estimated proportion and ground-truth proportion in the benchmark dataset GSE107011. Each row represents deconvolution method, (A) GLDADec, (B) GTM-decon, (C) BayesPrism, (D) CIBERSORTx, (E) MuSiC, and (F) BSEQ-sc, respectively.

# **Figure S6**

**
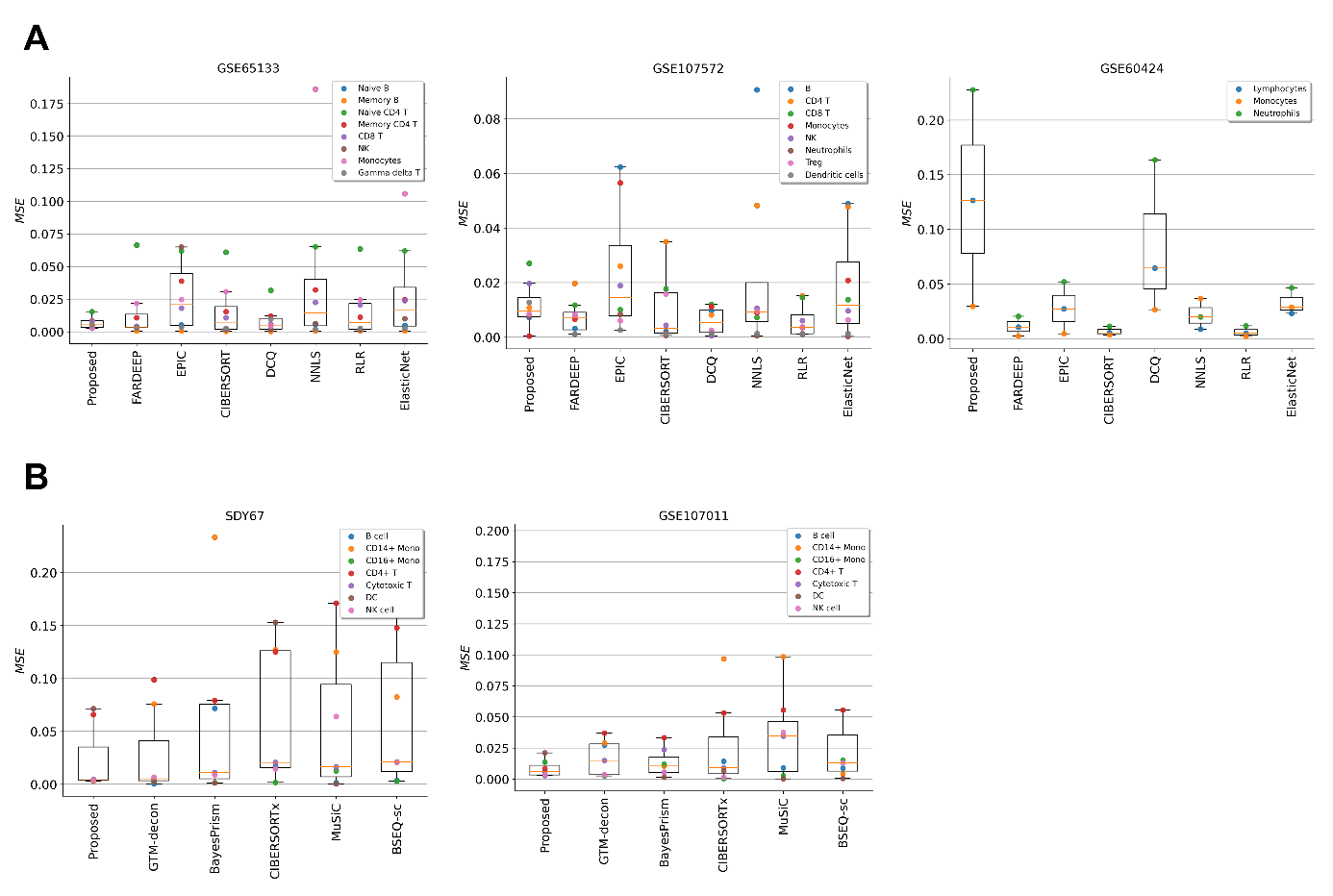
**

**Figure S6.** (A) Boxplots showing the mean square error (MSE) to compare the estimation performance against existing bulk reference-based methods using LM22 on three benchmark datasets, GSE65133, GSE107572, and GSE60424. (B) Boxplots showing the mean square error (MSE) to compare the estimation performance against the proposed method to state-of-the-art methods on two benchmark datasets, SDY67 and GSE107011. Each box extends from the 25^th^ percentile (bottom) to 75^th^ percentile (top), and the whisker indicates the farthest data point within 1.5-fold of inter-quartile range.

# **Figure S7**

**
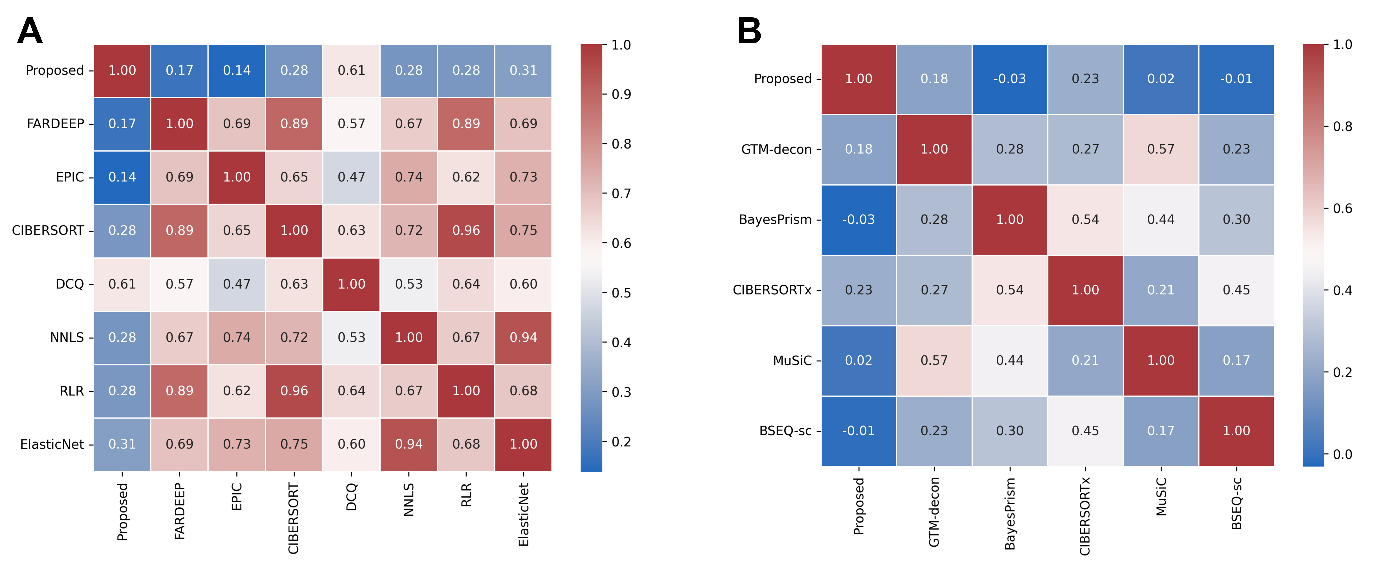
**

**Figure S7.** Similarity matrix of estimated values among (A) traditional bulk reference-based methods and (B) state-of-the-art methods.

# **Figure S8**

**
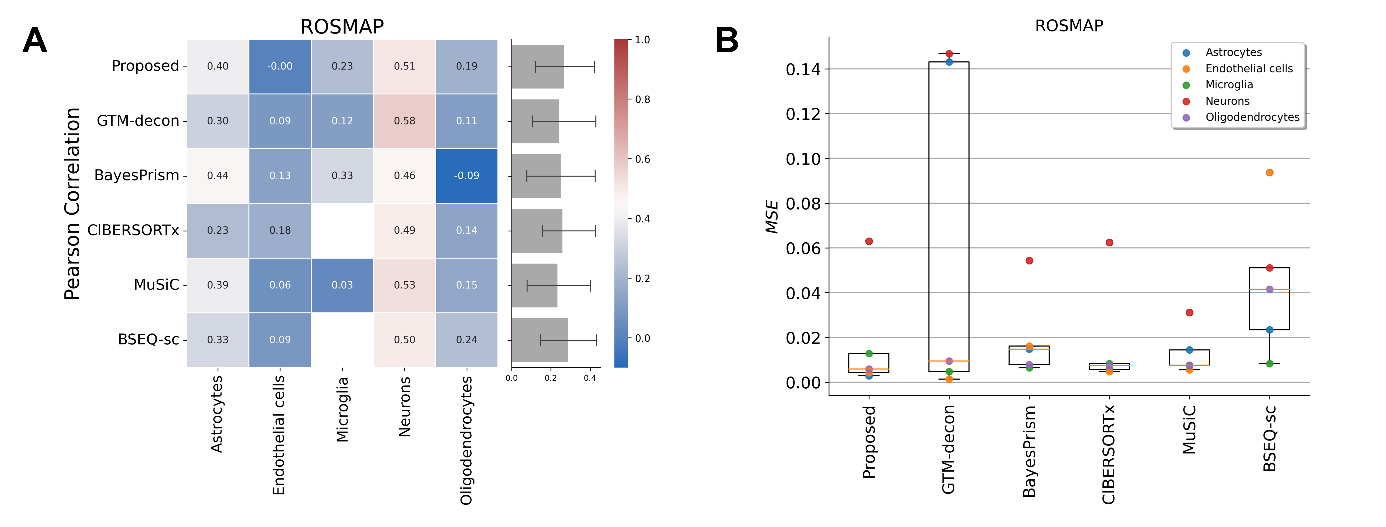
**

**Figure S8.** Benchmarking with human brain prefrontal cortex bulk dataset. (A) Heatmaps of comparing the estimation performance against state-of-the-art methods for Pearson correlation. The barplots on the right shows the performance of each method across all cell types. (B) Boxplots showing the mean square error (MSE) to compare the estimation performance against the proposed method to state-of-the-art methods. Each box extends from the 25^th^ percentile (bottom) to 75^th^ percentile (top), and the whisker indicates the farthest data point within 1.5-fold of inter-quartile range.

# **Figure S9**

**
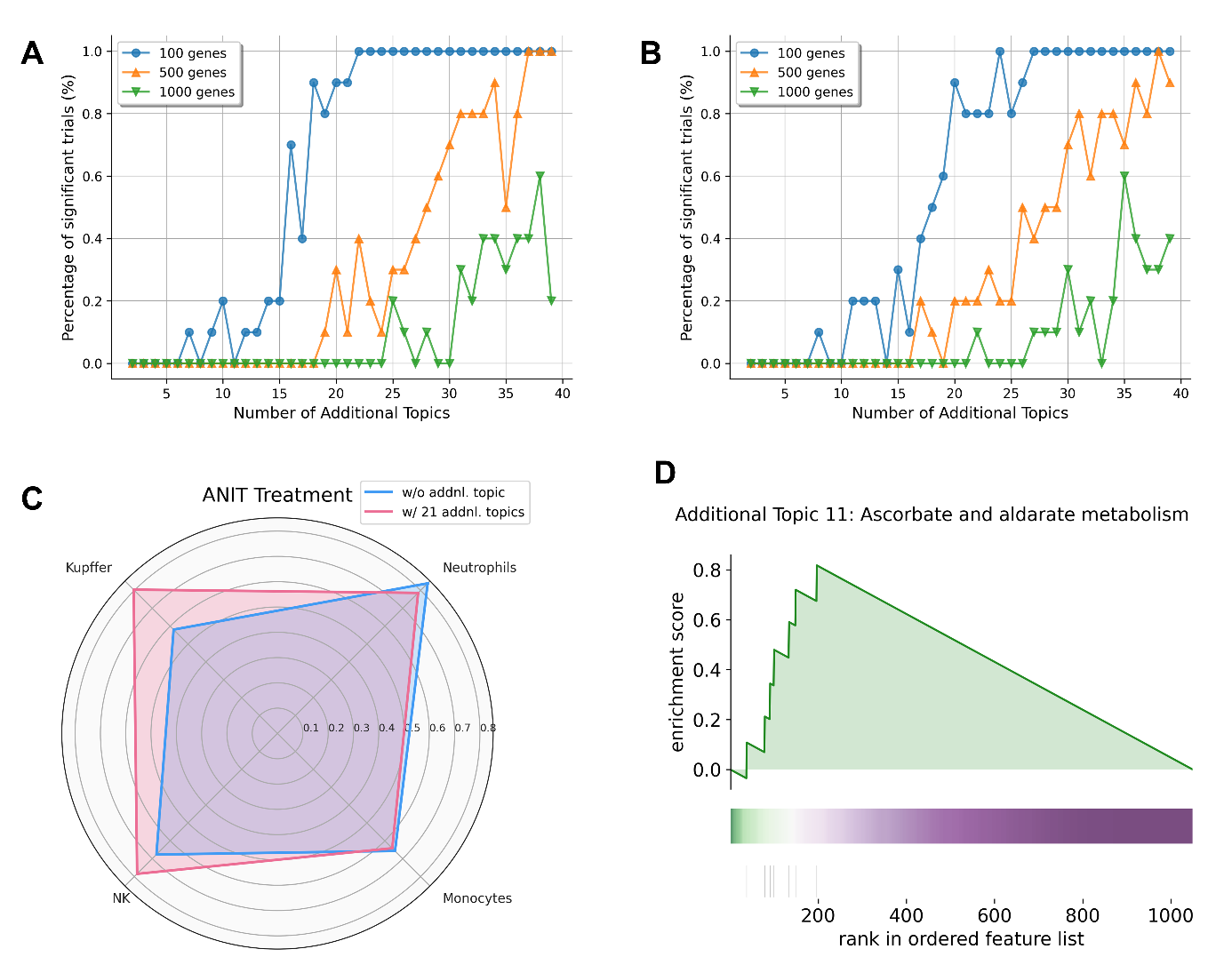
**

**Figure S9.** Evaluation of the usefulness of additional topics in the analysis of perturbed tissue data. The relationship between the number of additional topics that are significantly independent of each other and the number of genes to be analyzed for (A) acetaminophen (APAP)-perturbed and (B) alpha-naphthyl isothiocyanate (ANIT)-perturbed liver tissue. (C) A Radar chart comparing the difference in estimated performance with and without the additional topics for ANIT-perturbed liver tissue. The axis values indicate the Pearson correlation between estimated and measured proportion of each immune cells. (D) Gene set enrichment analysis (GSEA) was carried out on the ranked list of genes ranked according to their contribution to Topic 11. The colored band signifies the extent of each gene's contribution to the additional topic 11, with green representing a high contribution and purple indicating a low contribution. The bottom vertical black lines indicate the position of the genes that overlap with the pathway.

# **Figure S10**

**
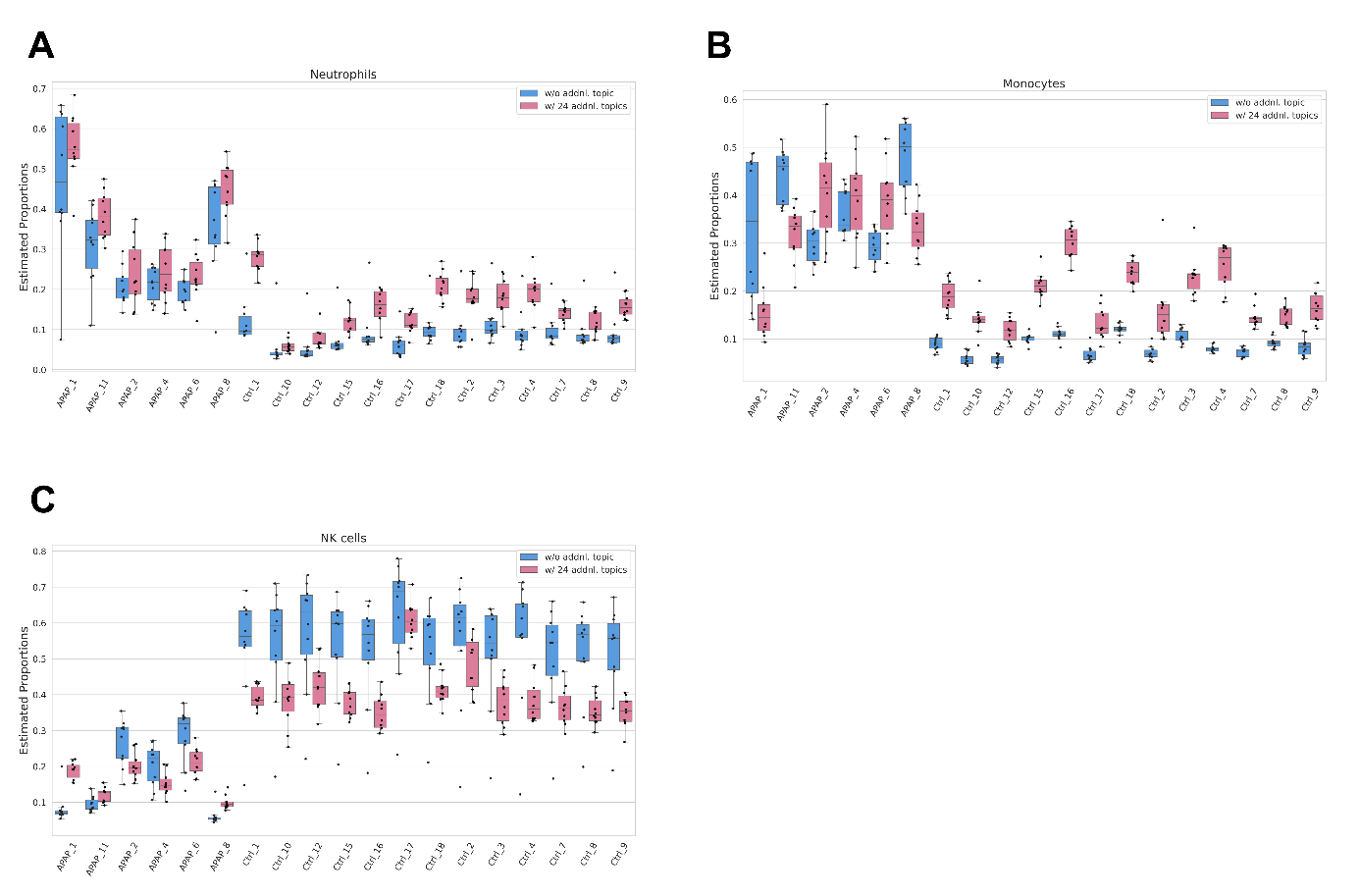
**

**Figure S10.** Variance of the estimate at ensemble in each sample with and without additional topics considered. Bar plots showing the estimated values of **(A)** neutrophils, **(B)** monocytes, and **(C**) Natural killer cells in each sample after APAP administration.

# **Figure S11**

**
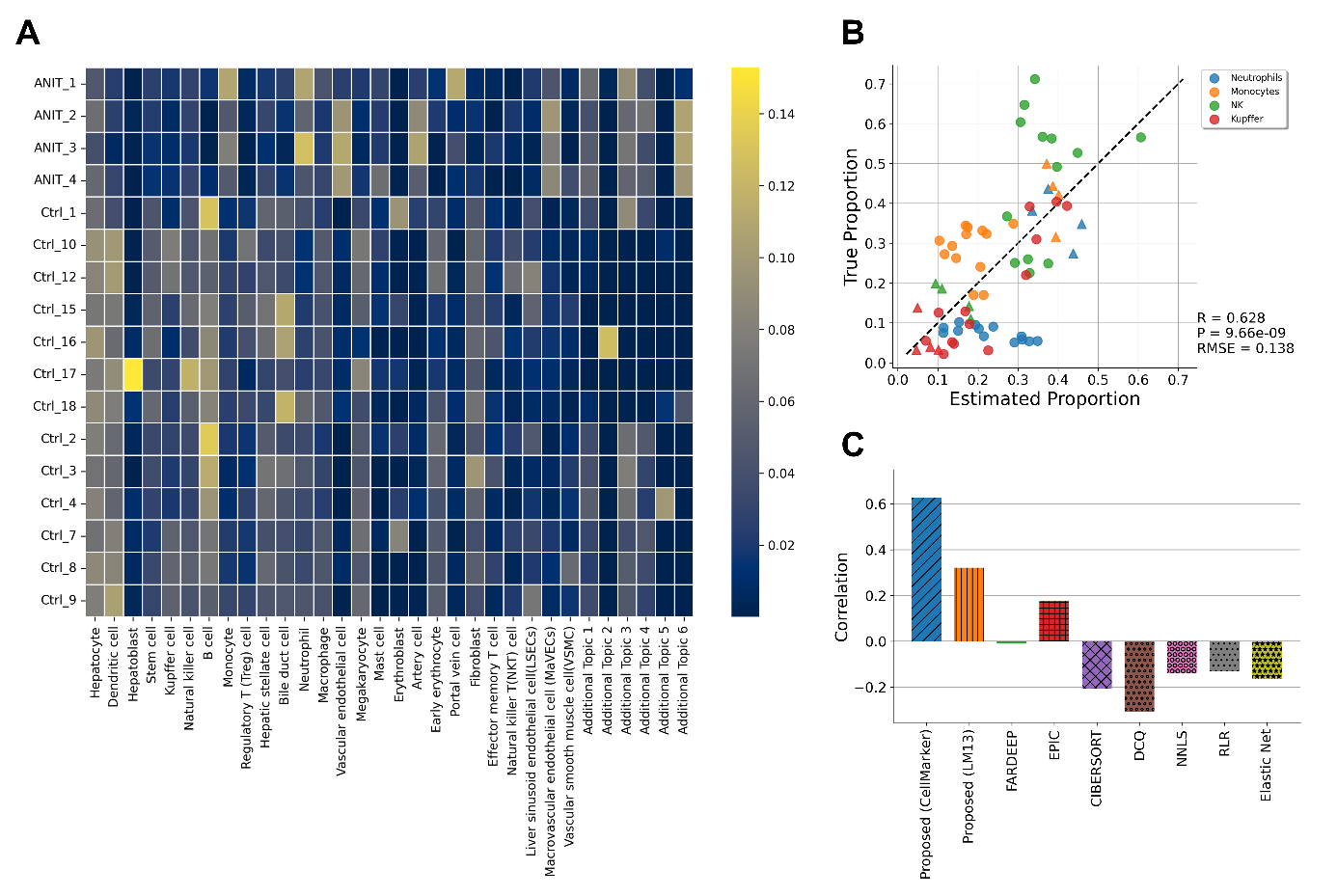
**

**Figure S11.** Prediction of immune cell trafficking in mouse liver tissue perturbed by alpha-naphthyl isothiocyanate (ANIT). **(A)** Comprehensive cell type proportions estimation using marker gene names collected in data-driven manner. **(B)** Scatterplot showing the estimated proportions of immune cells vs. measured values in the same dataset. Circles indicate control samples and triangles indicate samples treated with ANIT. **(C)** Performance comparison based on Pearson correlation with other notable deconvolution methods.

# **Figure S12**

**
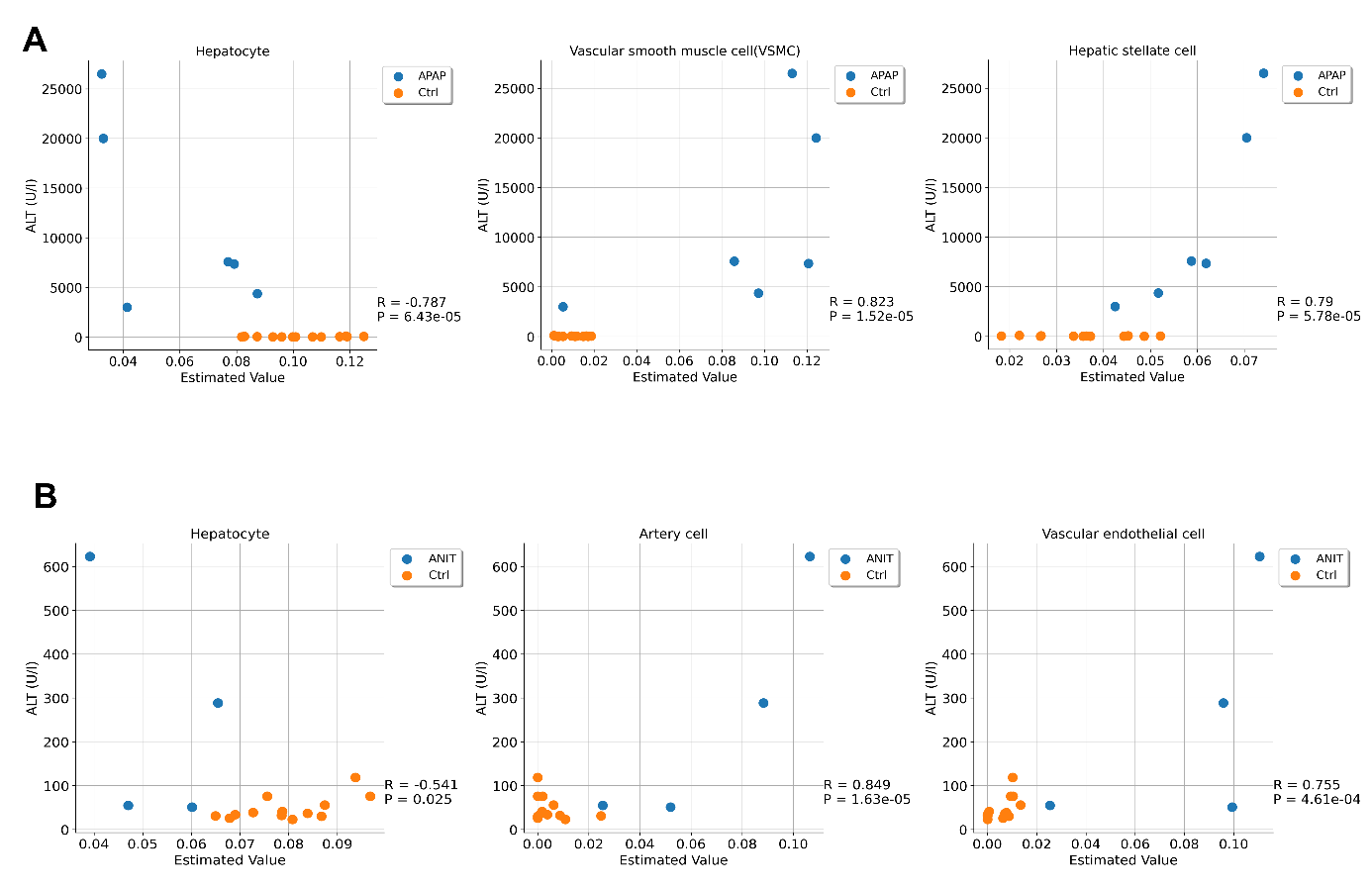
**

**Figure S12.** Relationship between the cell ratio estimated by GLDADec and the degree of injury. Scatterplots comparing estimated proportions and blood biochemistry value in **(A)** APAP and **(B)** ANIT treatment group, respectively. Alanine aminotransferase (ALT) is the most common marker of liver damage. High ALT values are a sign of severe liver damage.

# **Figure S13**

**
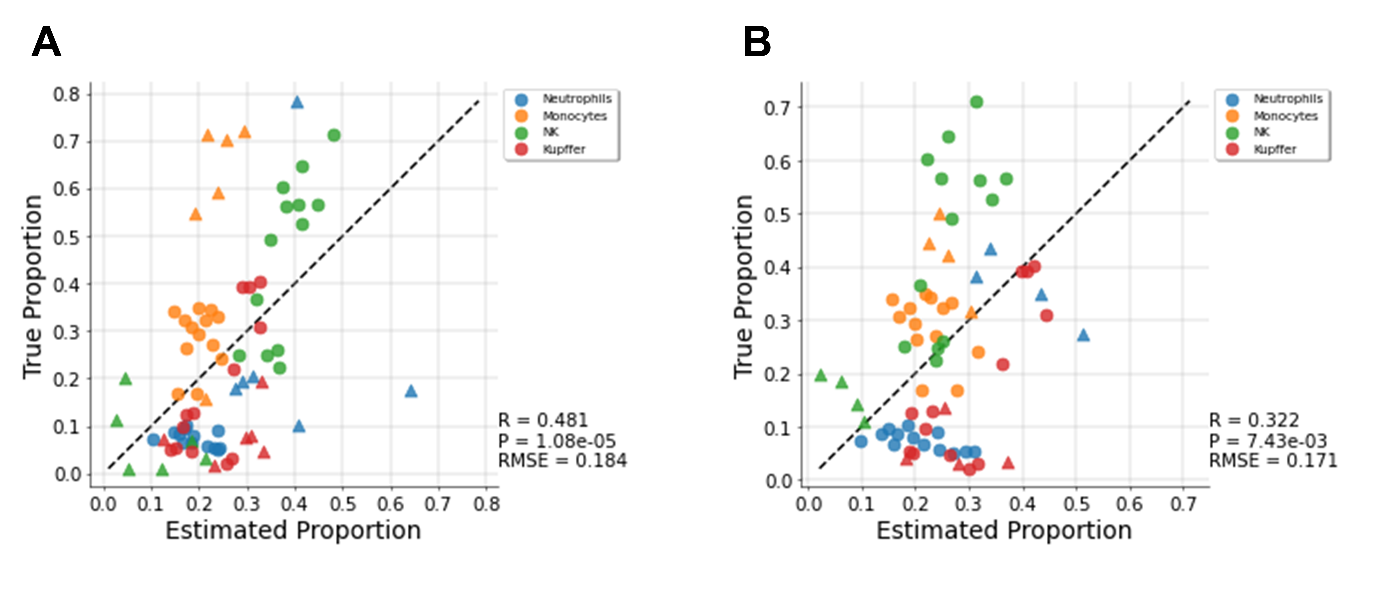
**

**Figure S13.** Estimation performance of GLDADec when using differentially expressed genes (DEGs) derived marker genes as prior information. Scatterplot showing the estimated proportions of immune cells vs. measured values for **(A)** acetaminophen and **(B)** alpha-naphthyl isothiocyanate administration group, respectively.

# **Figure S14**

**
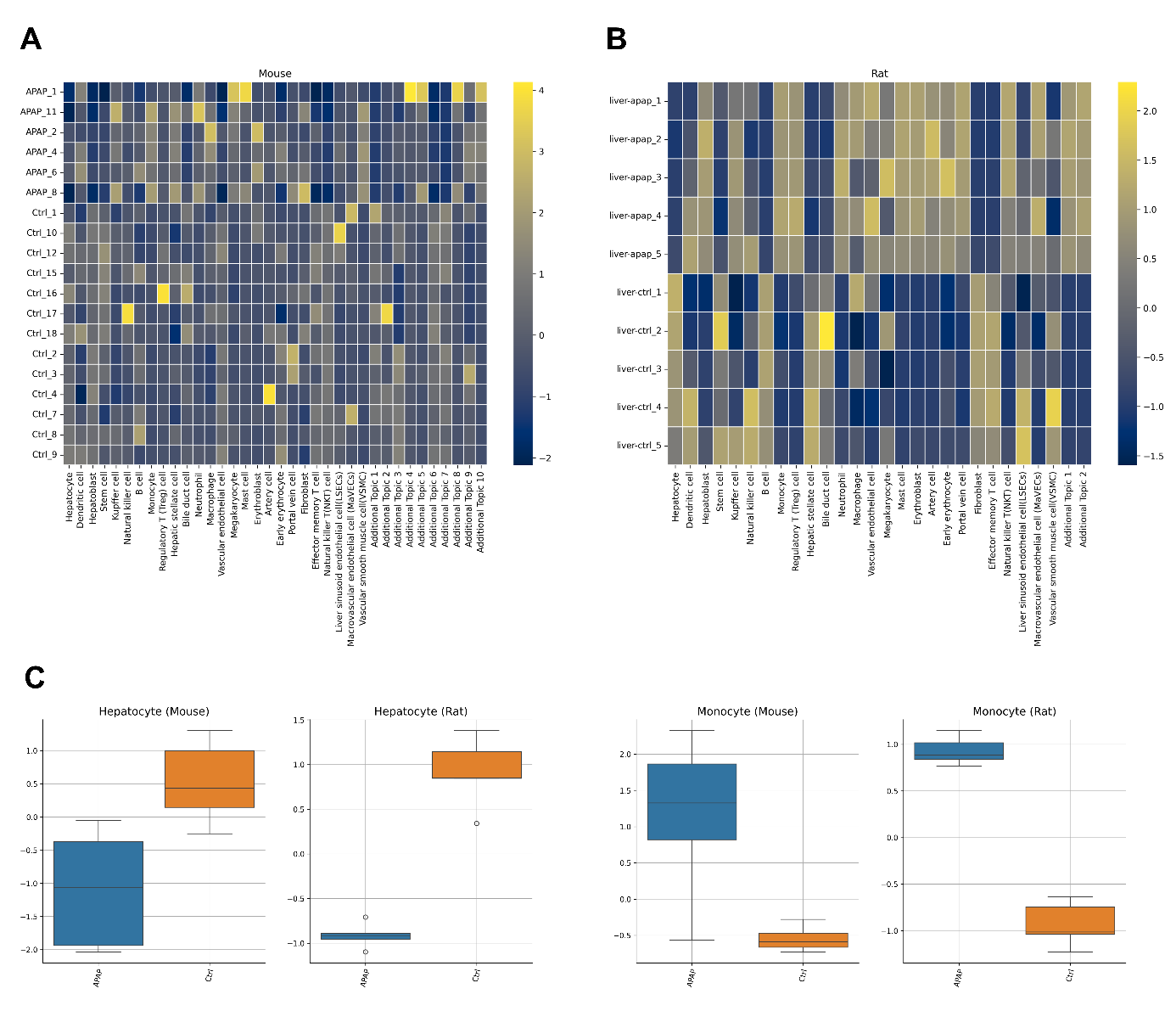
**

**Figure S14.** Comparison of immune cell trafficking patterns in mice and rats after acetaminophen (APAP) administration. Heatmaps showing the estimated comprehensive cell type proportions in **(A)** mice and **(B)** rats. **(C)** Common cell trafficking patterns between mice and rats.

# **Figure S15**

**
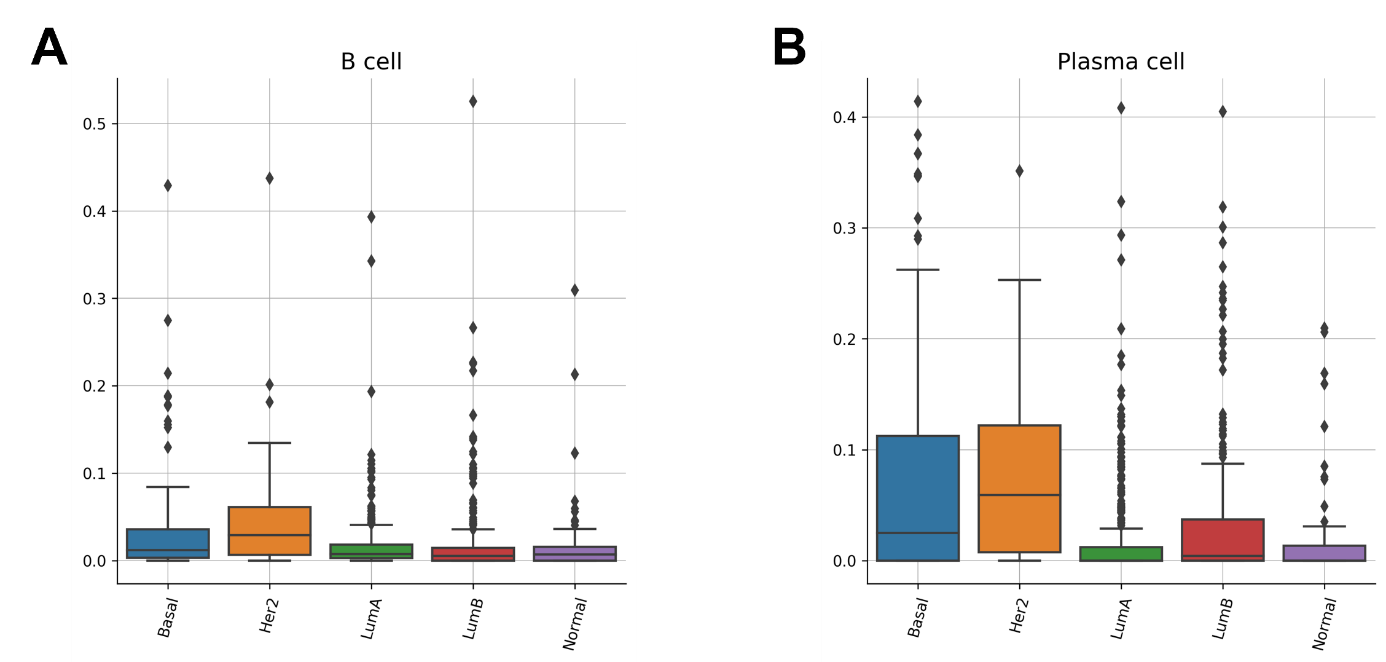
**

**Figure S15.** Boxplots showing specific accumulation and infiltration between each subtype of BRCA. Her2 subtype-specific accumulation was observed in **(A)** B cells and **(B)** plasma cells, respectively.

# **Figure S16**

**
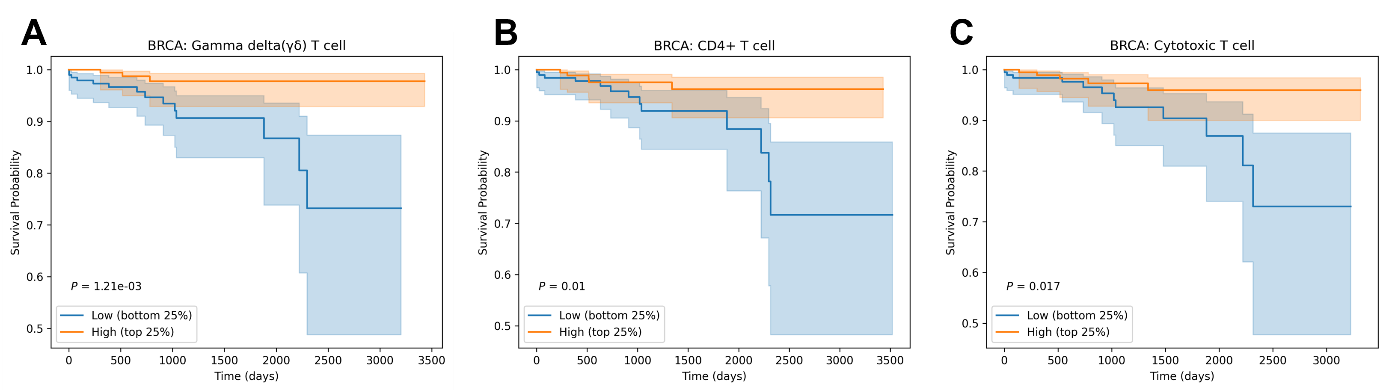
**

**Figure S16.** Survival analysis when samples are stratified by the accumulation of cells estimated by GLDADec. Kaplan-Meier plots showing survival associations with infiltration of **(A)** gamma delta (γδT) cells, **(B)** CD4+ T cells, and **(C)** Cytotoxic T cells in BRCA. Patients with the top 25^th^ percentile of the target cells were compared with those with the bottom 25^th^ percentile.

# **Figure S17**

**
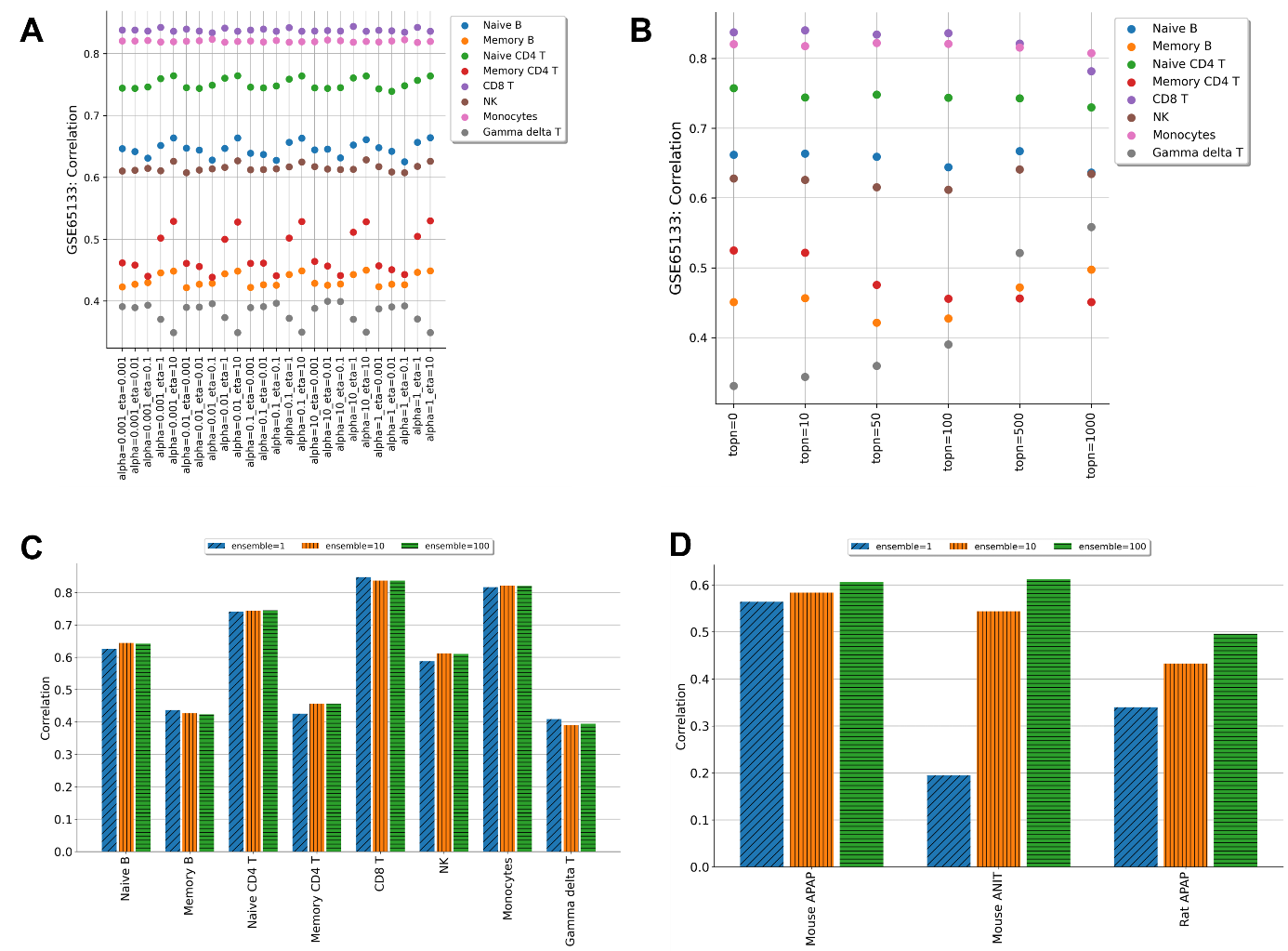
**

**Figure S17.** Hyperparameter sensitivity analysis. **(A)** Effect of the combination of hyperparameters α and η on the Dirichlet distributions. **(B)** Relationship between the number of additional genes with large coefficients of variation and estimation performance. Bar plots representing the contribution of the ensemble to improved estimation performance for **(C)** blood-derived samples and **(D)** tissue samples, respectively.

# **Figure S18**

**
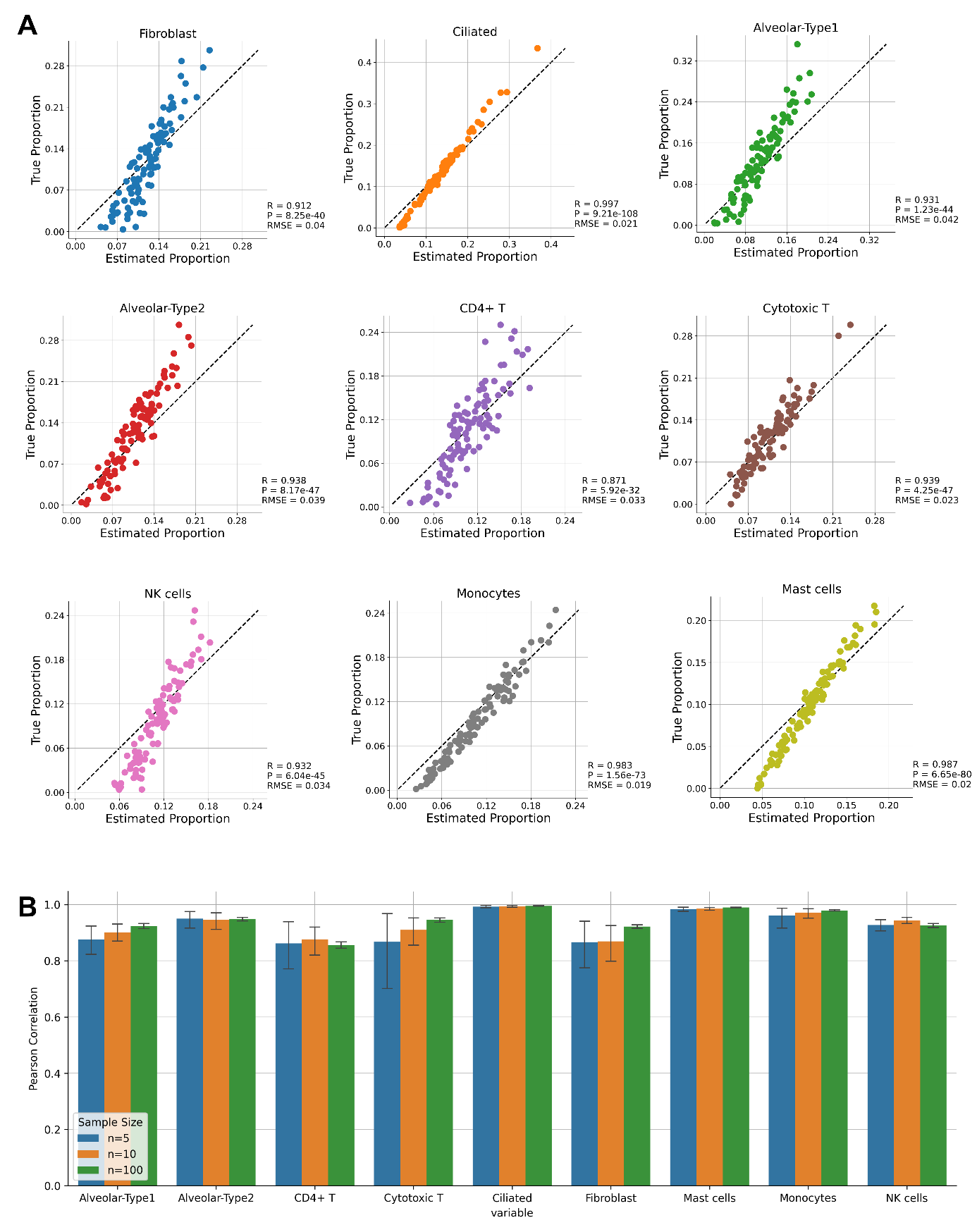
**

**Figure S18.** Assessing the robustness of the estimation performance of GLDADec. (A) Scatterplots showing estimated and ground-truth proportion for pseudo lung tissue data generated from single cell RNA-Seq. We generated 100 samples with randomly assigned proportions for the nine cell types. (B) Barplots showing the change in estimation performance with decreasing the number of samples.

# **Figure S19**

**
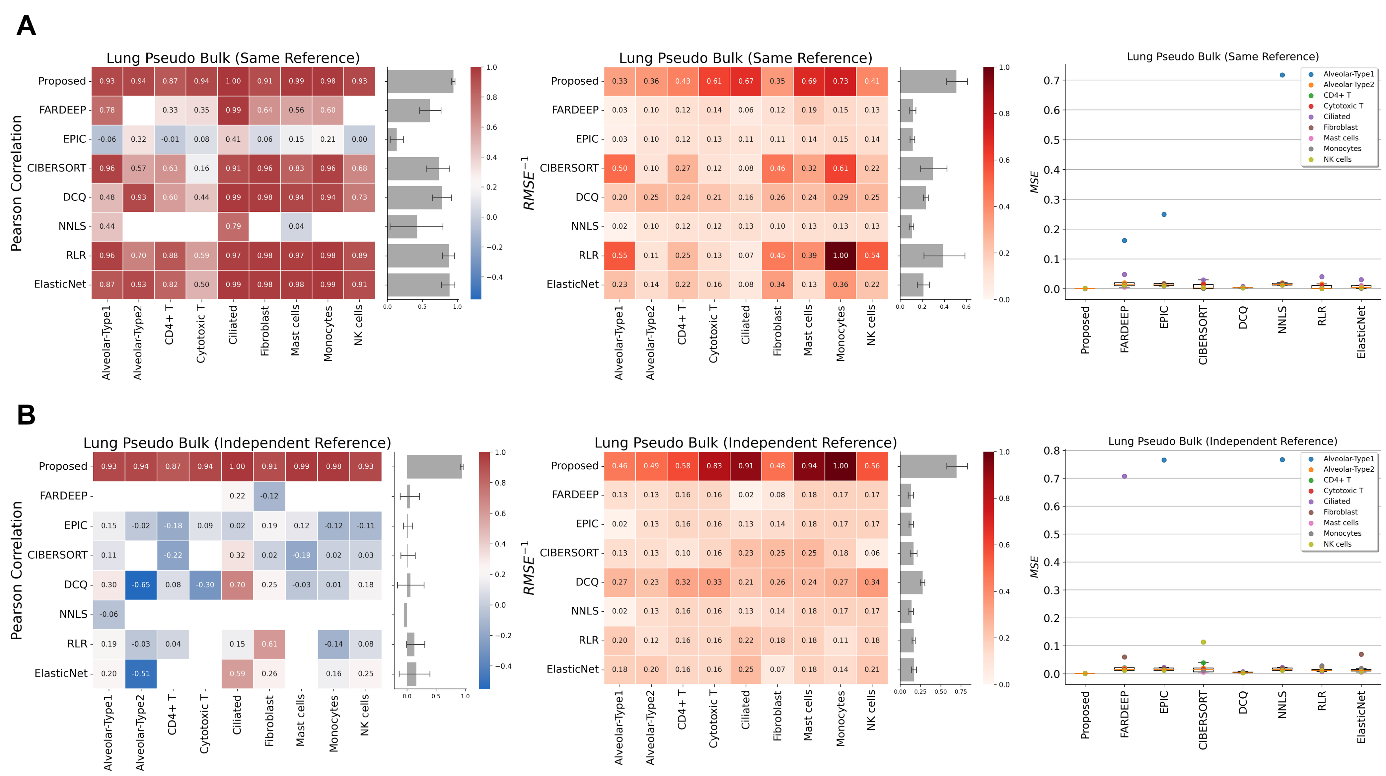
**

**Figure S19.** Assessing the robustness of GLDADec that is independent of the distribution of reference expression levels. Heatmap showing estimation performance when using a reference from (A) the same dataset and (B) an independent dataset. The left and center blocks show the Pearson correlation and the inversed root mean square error (RMSE) values (scaled 0 to 1), respectively. The barplots on the right shows the performance of each method across all cell types. The right block shows boxplots of mean square error (MSE). Each box extends from the 25^th^ percentile (bottom) to 75^th^ percentile (top), and the whisker indicates the farthest data point within 1.5-fold of inter-quartile range.

**Supplementary tables**

# **Table S1**

**Table S1.** Summary of datasets used in this study.

| **Dataset** | **PMID** | **Size** | **Species** | **Source** |
| --- | --- | --- | --- | --- |
| GSE65133 | 25822800 | 20 | Human | PBMC |
| GSE107572 | 31126321 | 9 | Human | PBMC/PMN |
| GSE60424 | 25314013 | 5 | Human | Whole blood |
| SDY67 | 27031986 | 267 | Human | PBMC |
| GSE107011 | 30726743 | 12 | Human | PBMC |
| ROSMAP | 32804935 | 41 | Human | Brain |
| GSE237801-APAP | 38187088 | 19 | Mouse | Liver |
| GSE237801-ANIT | 38187088 | 17 | Mouse | Liver |
| GSE239996-APAP | 37941435 | 10 | Rat | Liver |
| TCGA-BRCA | - | 1052 | Human | Breast |
| TCGA-LUAD | - | 577 | Human | Lung |
| TCGA-LIHC | - | 408 | Human | Liver |
| Tissue Stability Cell Atlas | 31892341 | - | Human | Lung (single cell) |
| GSE131907 | 32385277 | - | Human | Lung (single cell) |

# **Table S2**

**Table S2.** Gene Ontology (GO) enrichment analysis for the added topic that reconstructing missing neuron. The top 10 significantly enriched GO terms are shown with Benjamini–Hochberg adjusted p-values.

| Rank | GO Term | Adjusted P-value | Overlap |
| --- | --- | --- | --- |
| 1 | Chemical Synaptic Transmission (GO:0007268) | 0.001153 | {*'GABRA1', 'SST', 'SNAP25', 'GAD2', 'GABRD', 'SLC1A1', 'SLC17A7', 'GAD1', 'SLC17A6'*} |
| 2 | L-glutamate Import (GO:0051938) | 0.009144 | {*'SLC17A6', 'SLC1A1', 'SLC17A7'*} |
| 3 | Neurotransmitter Transport (GO:0006836) | 0.01298 | {*'SNAP25', 'SLC17A6', 'SLC1A1', 'SLC17A7'*} |
| 4 | Cellular Response To Nerve Growth Factor Stimulus (GO:1990090) | 0.01313 | {*'MAPT', 'STMN2', 'SH3GL2'*} |
| 5 | Positive Regulation Of Microtubule Polymerization Or Depolymerization (GO:0031112) | 0.01362 | {*'MAPT', 'STMN2', 'PAK1'*} |
| 6 | Glutamate Catabolic Process (GO:0006538) | 0.01362 | {*'GAD2', 'GAD1'*} |
| 7 | Gamma-Aminobutyric Acid Metabolic Process (GO:0009448) | 0.01362 | {*'GAD2', 'GAD1'*} |
| 8 | Anterograde Trans-Synaptic Signaling (GO:0098916) | 0.01362 | {*'SNAP25', 'SST', 'GAD2', 'GABRD', 'SLC1A1', 'GAD1'*} |
| 9 | Sodium-Dependent Phosphate Transport (GO:0044341) | 0.02114 | {*'SLC17A6', 'SLC17A7'*} |
| 10 | Regulation Of Mesenchymal Stem Cell Differentiation (GO:2000739) | 0.02114 | {*'PDGFRA', 'SOX6'*} |

# **Table S3**

**Table S3.** Gene ontology (GO) enrichment analysis. The top 10 significantly enriched GO terms are shown with Benjamini–Hochberg adjusted *P*-values.

| Topics | Gene Ontology | Adjusted *P*-value | Overlap |
| --- | --- | --- | --- |
| 4 | Sterol Biosynthetic Process (GO:0016126) | 0.001719 | *{'Nsdhl', 'Sqle', 'Pmvk', 'Hsd17b7', 'Fdft1'}* |
|  | Secondary Alcohol Biosynthetic Process (GO:1902653) | 0.008193 | *{'Nsdhl', 'Fdft1', 'Hsd17b7', 'Pmvk'}* |
|  | Cholesterol Biosynthetic Process (GO:0006695) | 0.008193 | *{'Nsdhl', 'Fdft1', 'Hsd17b7', 'Pmvk'}* |
|  | Cholesterol Metabolic Process (GO:0008203) | 0.03142 | *{'Nsdhl', 'Sqle', 'Pmvk', 'Hsd17b7', 'Fdft1'}* |
|  |  |  |  |
| 6 | Lipid Biosynthetic Process (GO:0008610) | 0.0001040 | *{'Srd5a1', 'Mvd', 'Cyp17a1', 'Mvk', 'Acaca', 'Acacb', 'Acsm5', 'Fitm1', 'Fdft1', 'Gpat4'}* |
|  | Secondary Alcohol Biosynthetic Process (GO:1902653) | 0.009539 | *{'Tm7sf2', 'Mvd', 'Mvk', 'Pmvk', 'Fdft1'}* |
|  | Cholesterol Biosynthetic Process (GO:0006695) | 0.009539 | *{'Tm7sf2', 'Mvd', 'Mvk', 'Pmvk', 'Fdft1'}* |
|  | Sterol Biosynthetic Process (GO:0016126) | 0.01275 | *{'Tm7sf2', 'Mvd', 'Mvk', 'Pmvk', 'Fdft1'}* |
|  | acetyl-CoA Metabolic Process (GO:0006084) | 0.01443 | *{'PmvK', 'Mvd', 'Mvk', 'Acacb'}* |
|  |  |  |  |
| 11 | Lipid Biosynthetic Process (GO:0008610) | 0.01496 | *{'Acsm3', 'Srd5a1', 'Prlr', 'Mvk', 'Acaca', 'Acacb', 'Acsm5', 'Fitm1', 'Fdft1', 'Gpat4'}* |
|  | Glucan Biosynthetic Process (GO:0009250) | 0.01927 | *{'Gys2', 'Ppp1r3c', 'Ugp2', 'Nr1d1'}* |
|  | Glycogen Biosynthetic Process (GO:0005978) | 0.01927 | *{'Gys2', 'Ppp1r3c', 'Ugp2', 'Nr1d1'}* |
|  | Monocarboxylic Acid Catabolic Process (GO:0072329) | 0.03638 | *{'Ppard', 'Cyp26a1', 'Agxt2', 'Faah', 'Lpin1'}* |
|  |  |  |  |
| 14 | Response To Lipopolysaccharide (GO:0032496) | 0.00001500 | *{'Selp', 'Zc3h12a', 'Nfkbib', 'Tnfaip3', 'Mapkapk2', 'Pde4b', 'Sele', 'Pf4', 'Cxcl3', 'Il1a', 'Irak3', 'Tnip1', 'Sbno2', 'Cd274', 'Tnip2'}* |
|  | Cellular Response To Lipopolysaccharide (GO:0071222) | 0.0001779 | *{'Zc3h12a', 'Nfkbib', 'Tnfaip3', 'Pde4b', 'Pf4', 'Cxcl3', 'Il1a', 'Tnip1', 'Sbno2', 'Cd274', 'Tnip2', 'Ccl3'}* |
|  | Response To Interleukin-1 (GO:0070555) | 0.0001988 | *{'Zc3h12a', 'Ccl19', 'Irak2', 'Sele', 'Irak3', 'Sox9', 'Ccl7', 'Ccl3', 'Ccl4', 'Src'}* |
|  | Cellular Response To Molecule Of Bacterial Origin (GO:0071219) | 0.0003834 | *{'Zc3h12a', 'Nfkbib', 'Tnfaip3', 'Pde4b', 'Pf4', 'Cxcl3', 'Il1a', 'Tnip1', 'Sbno2', 'Cd274', 'Tnip2'}* |
|  | Response To Cytokine (GO:0034097) | 0.0005927 | *{'Ifitm1', 'Selp', 'Csf3', 'Timp1', 'Irak2', 'Mapkapk2', 'Sele', 'Irak3', 'Sphk1', 'Cd274', 'Src'}* |
|  | Cellular Response To Interleukin-1 (GO:0071347) | 0.0006260 | *{'Zc3h12a', 'Ccl19', 'Irak2', 'Irak3', 'Sox9', 'Ccl7', 'Ccl4', 'Tnip2', 'Ccl3'}* |
|  | Cytokine-Mediated Signaling Pathway (GO:0019221) | 0.001083 | *{'Csf3', 'Ccl19', 'Irak2', 'Il17ra', 'Pf4', 'Cxcl3', 'Il1a', 'Ccl4', 'Ccl7', 'Socs1', 'Ccl3', 'Irak3', 'Lepr', 'Tnip2', 'Src'}* |
|  | Cellular Response To Lipid (GO:0071396) | 0.001106 | *{'Zc3h12a', 'Nfkbib', 'Tnfaip3', 'Pde4b', 'Pf4', 'Sox9', 'Hspa1b', 'Il1a', 'Hspa1a', 'Cxcl3', 'Tnip1', 'Sbno2', 'Cd274', 'Tnip2'}* |
|  | Cellular Response To Chemokine (GO:1990869) | 0.003317 | *{'Zc3h12a', 'Ccl19', 'Pf4', 'Cxcl3', 'Ccl7', 'Ccl4', 'Ccl3'}* |
|  | Cellular Response To Heat (GO:0034605) | 0.003317 | *{'Ier5', 'Dnajb1', 'Hspa1b', 'Il1a', 'Hspa1a', 'Bag3'}* |
|  |  |  |  |
| 21 | Response To Lipopolysaccharide (GO:0032496) | 0.001879 | *{'Selp', 'Zc3h12a', 'Nfkbib', 'Cxcl13', 'Tnfaip3', 'Mapkapk2', 'Pde4b', 'Sele', 'Pf4', 'Cxcl3', 'Il1a', 'Sbno2', 'Tnip2'}* |
|  | Cellular Response To Chemokine (GO:1990869) | 0.001916 | *{'Zc3h12a', 'Ccl19', 'Cxcl13', 'Pf4', 'Cxcl3', 'Ccl7', 'Ccl4', 'Ccl3'}* |
|  | Cellular Response To Lipopolysaccharide (GO:0071222) | 0.001916 | *{'Zc3h12a', 'Nfkbib', 'Cxcl13', 'Tnfaip3', 'Pde4b', 'Pf4', 'Cxcl3', 'Il1a', 'Sbno2', 'Tnip2', 'Ccl3'}* |
|  | Cytokine-Mediated Signaling Pathway (GO:0019221) | 0.003719 | *{'Mt3', 'Ccl19', 'Irak2', 'Cxcl13', 'Il17ra', 'Pf4', 'Birc3', 'Il1a', 'Ccl4', 'Cxcl3', 'Socs1', 'Ccl7', 'Lepr', 'Tnip2', 'Ccl3'}* |
|  | Neutrophil Chemotaxis (GO:0030593) | 0.003719 | *{'Ccl19', 'Cxcl13', 'Pde4b', 'Pf4', 'Cxcl3', 'Ccl7', 'Ccl4', 'Ccl3'}* |
|  | Cellular Response To Molecule Of Bacterial Origin (GO:0071219) | 0.003719 | *{'Zc3h12a', 'Nfkbib', 'Cxcl13', 'Tnfaip3', 'Pde4b', 'Pf4', 'Cxcl3', 'Il1a', 'Sbno2', 'Tnip2'}* |
|  | Granulocyte Chemotaxis (GO:0071621) | 0.003847 | *{'Ccl19', 'Cxcl13', 'Pde4b', 'Pf4', 'Cxcl3', 'Ccl7', 'Ccl4', 'Ccl3'}* |
|  | Neutrophil Migration (GO:1990266) | 0.004618 | *{'Ccl19', 'Cxcl13', 'Pde4b', 'Pf4', 'Cxcl3', 'Ccl7', 'Ccl4', 'Ccl3'}* |
|  | Chemokine-Mediated Signaling Pathway (GO:0070098) | 0.004618 | *{'Ccl19', 'Cxcl13', 'Pf4', 'Cxcl3', 'Ccl7', 'Ccl4', 'Ccl3'}* |
|  | ERK1 And ERK2 Cascade (GO:0070371) | 0.004618 | *{'Mt3', 'Dusp6', 'Myc', 'Dusp5', 'Sox9'}* |
|  |  |  |  |
| 22 | Sterol Biosynthetic Process (GO:0016126) | 0.00001234 | *{'Nsdhl', 'Sqle', 'Mvd', 'Mvk', 'Ch25h', 'Pmvk', 'Hsd17b7', 'Fdft1'}* |
|  | Secondary Alcohol Biosynthetic Process (GO:1902653) | 0.0009564 | *{'Nsdhl', 'Mvd', 'Mvk', 'Pmvk', 'Hsd17b7', 'Fdft1'}* |
|  | Cholesterol Biosynthetic Process (GO:0006695) | 0.0009564 | *{'Nsdhl', 'Mvd', 'Mvk', 'Pmvk', 'Hsd17b7', 'Fdft1'}* |
|  | Cholesterol Metabolic Process (GO:0008203) | 0.003749 | *{'Nsdhl', 'Sqle', 'Mvd', 'Mvk', 'Ch25h', 'Pmvk', 'Hsd17b7', 'Fdft1'}* |
|  |  |  |  |
| 23 | Response To Interleukin-1 (GO:0070555) | 0.00001373 | *{'Rela', 'Gbp2', 'Zc3h12a', 'Irak2', 'Sele', 'Hif1a', 'Sox9', 'Ccl7', 'Ccl3', 'Irak3', 'Nr1d1', 'Ccl4', 'Src'}* |
|  | Response To Lipopolysaccharide (GO:0032496) | 0.00001373 | *{'Rela', 'Gbp2', 'Selp', 'Zc3h12a', 'Nfkbib', 'Cxcl13', 'Tnfaip3', 'Mapkapk2', 'Pde4b', 'Sele', 'Pf4', 'Irak3', 'Nr1d1', 'Tnip1', 'Sbno2', 'Cd274', 'Tnip2'}* |
|  | Cellular Response To Interleukin-1 (GO:0071347) | 0.00003392 | *{'Rela', 'Gbp2', 'Zc3h12a', 'Irak2', 'Hif1a', 'Sox9', 'Ccl7', 'Irak3', 'Nr1d1', 'Ccl4', 'Tnip2', 'Ccl3'}* |
|  | Cellular Response To Lipopolysaccharide (GO:0071222) | 0.00006671 | *{'Rela', 'Gbp2', 'Zc3h12a', 'Nfkbib', 'Cxcl13', 'Tnfaip3', 'Pde4b', 'Pf4', 'Nr1d1', 'Tnip1', 'Sbno2', 'Cd274', 'Tnip2', 'Ccl3'}* |
|  | Cellular Response To Molecule Of Bacterial Origin (GO:0071219) | 0.0001809 | *{'Rela', 'Gbp2', 'Zc3h12a', 'Nfkbib', 'Cxcl13', 'Tnfaip3', 'Pde4b', 'Pf4', 'Nr1d1', 'Tnip1', 'Sbno2', 'Cd274', 'Tnip2'}* |
|  | Response To Cytokine (GO:0034097) | 0.0003242 | *{'Rela', 'Ifitm1', 'Selp', 'Timp1', 'Timp3', 'Irak2', 'Mapkapk2', 'Sele', 'Irak3', 'Isg15', 'Sphk1', 'Cd274', 'Src'}* |
|  | Cellular Response To Lipid (GO:0071396) | 0.0007037 | *{'Rela', 'Pdk4', 'Gbp2', 'Zc3h12a', 'Nfkbib', 'Cxcl13', 'Tnfaip3', 'Pde4b', 'Pf4', 'Sox9', 'Hspa1b', 'Hspa1a', 'Nr1d1', 'Tnip1', 'Sbno2', 'Cd274', 'Tnip2'}* |
|  | ERK1 And ERK2 Cascade (GO:0070371) | 0.001265 | *{'Mt3', 'Dusp6', 'Myc', 'Zfp36l2', 'Dusp5', 'Sox9'}* |
|  | Regulation Of Tumor Necrosis Factor-Mediated Signaling Pathway (GO:0010803) | 0.007982 | *{'Tnfaip3', 'Mapkapk2', 'Hspa1b', 'Sphk1', 'Ppp2cb', 'Hspa1a', 'Ptpn2'}* |
|  | Response To Tumor Necrosis Factor (GO:0034612) | 0.009587 | *{'Rela', 'Gbp2', 'Zc3h12a', 'Zfp36l2', 'Sele', 'Ccl7', 'Sphk1', 'Nr1d1', 'Ccl4', 'Ccl3'}* |

# **Table S4**

**Table S4.**  Pearson correlation between cell proportions estimated by GLDADec and various liver injury markers such as ALT, AST, and TBIL after acetaminophen (APAP) administration.

| APAP | | | |
| --- | --- | --- | --- |
|  | ALT | AST | TBIL |
| ALT | 1.0000 | 0.9478 | 0.8273 |
| AST | 0.9478 | 1.0000 | 0.8868 |
| Monocyte | 0.9089 | 0.7421 | 0.6447 |
| Kupffer cell | 0.8743 | 0.7927 | 0.6921 |
| Neutrophil | 0.8644 | 0.7969 | 0.7706 |
| TBIL | 0.8273 | 0.8868 | 1.0000 |
| Vascular smooth muscle cell (VSMC) | 0.8228 | 0.6275 | 0.5233 |
| Hepatic stellate cell | 0.7898 | 0.6611 | 0.5469 |
| Fibroblast | 0.7503 | 0.7970 | 0.7505 |
| Additional Topic 5 | 0.5395 | 0.5439 | 0.6019 |
| Megakaryocyte | 0.5198 | 0.4101 | 0.4421 |
| Additional Topic 10 | 0.4709 | 0.3172 | 0.3690 |
| Additional Topic 8 | 0.4316 | 0.4543 | 0.5446 |
| Macrophage | 0.3874 | 0.1450 | 0.1044 |
| Mast cell | 0.3423 | 0.3707 | 0.4779 |
| Portal vein cell | 0.1883 | 0.2757 | 0.2154 |
| Erythroblast | 0.1010 | -0.1205 | -0.3088 |
| Dendritic cell | 0.0348 | -0.0410 | 0.0386 |
| Additional Topic 4 | -0.0234 | -0.0322 | 0.1259 |
| Artery cell | -0.1242 | -0.0959 | -0.1561 |
| Regulatory T (Treg) cell | -0.1324 | -0.0809 | 0.0167 |
| Macrovascular endothelial cell (MaVECs) | -0.1367 | -0.0674 | -0.0384 |
| Liver sinusoid endothelial cell (LSECs) | -0.1543 | -0.1708 | -0.3209 |
| Additional Topic 9 | -0.1746 | -0.2972 | -0.3245 |
| Bile duct cell | -0.1786 | -0.0540 | 0.0467 |
| Natural killer cell | -0.1866 | -0.1545 | 0.0449 |
| Additional Topic 2 | -0.2837 | -0.2044 | 0.0049 |
| B cell | -0.3217 | -0.3663 | -0.3739 |
| Early erythrocyte | -0.3922 | -0.4414 | -0.5754 |
| Additional Topic 3 | -0.4489 | -0.4141 | -0.5865 |
| Stem cell | -0.4723 | -0.3720 | -0.3441 |
| Additional Topic 1 | -0.6039 | -0.4434 | -0.4131 |
| Additional Topic 7 | -0.6698 | -0.4832 | -0.4904 |
| Effector memory T cell | -0.6759 | -0.6119 | -0.5993 |
| Vascular endothelial cell | -0.7522 | -0.6625 | -0.7224 |
| Natural killer T(NKT) cell | -0.7848 | -0.6279 | -0.6018 |
| Hepatoblast | -0.7868 | -0.6413 | -0.6707 |
| Hepatocyte | -0.7868 | -0.7143 | -0.7201 |
| Additional Topic 6 | -0.7870 | -0.6359 | -0.5908 |

# **Table S5**

**Table S5.** Pearson correlation between cell proportions estimated by GLDADec and various liver injury markers such as ALT, AST, and TBIL after alpha-naphthyl isothiocyanate (ANIT) administration.

| ANIT | | | |
| --- | --- | --- | --- |
|  | ALT | AST | TBIL |
| ALT | 1.0000 | 0.9837 | 0.8807 |
| AST | 0.9837 | 1.0000 | 0.9075 |
| TBIL | 0.8807 | 0.9075 | 1.0000 |
| Artery cell | 0.8490 | 0.8253 | 0.8658 |
| Vascular endothelial cell | 0.7549 | 0.7216 | 0.8763 |
| Additional Topic 6 | 0.7216 | 0.6840 | 0.8239 |
| Neutrophil | 0.6691 | 0.7145 | 0.7333 |
| Macrovascular endothelial cell (MaVECs) | 0.6394 | 0.5930 | 0.7701 |
| Monocyte | 0.4994 | 0.5086 | 0.6127 |
| Additional Topic 4 | 0.2371 | 0.2374 | 0.3291 |
| Additional Topic 1 | 0.2226 | 0.2758 | 0.4081 |
| Mast cell | 0.2084 | 0.1795 | 0.4790 |
| Portal vein cell | 0.2069 | 0.2387 | 0.3103 |
| Additional Topic 3 | 0.1772 | 0.2330 | 0.2685 |
| Effector memory T cell | -0.0085 | 0.0074 | -0.0951 |
| Additional Topic 2 | -0.0376 | -0.0676 | -0.0619 |
| Hepatoblast | -0.0565 | -0.0824 | -0.0485 |
| Natural killer T(NKT) cell | -0.0570 | -0.1021 | -0.2580 |
| Additional Topic 5 | -0.1332 | -0.1093 | -0.1711 |
| Erythroblast | -0.2500 | -0.1994 | -0.2383 |
| Natural killer cell | -0.2920 | -0.3137 | -0.3087 |
| Regulatory T (Treg) cell | -0.3018 | -0.3478 | -0.4882 |
| Kupffer cell | -0.3059 | -0.3175 | -0.4427 |
| Vascular smooth muscle cell (VSMC) | -0.3061 | -0.2617 | -0.1206 |
| Stem cell | -0.3683 | -0.4029 | -0.5304 |
| Liver sinusoid endothelial cell (LSECs) | -0.3801 | -0.3444 | -0.4674 |
| Bile duct cell | -0.4157 | -0.4109 | -0.4434 |
| Macrophage | -0.4244 | -0.4285 | -0.4181 |
| Early erythrocyte | -0.4520 | -0.4542 | -0.6601 |
| Megakaryocyte | -0.4991 | -0.4974 | -0.5964 |
| Fibroblast | -0.5285 | -0.5247 | -0.6216 |
| Hepatocyte | -0.5410 | -0.5995 | -0.7191 |
| Dendritic cell | -0.5531 | -0.5706 | -0.6903 |
| B cell | -0.5755 | -0.5371 | -0.6592 |
| Hepatic stellate cell | -0.5780 | -0.5506 | -0.6094 |

**Supplementary notes**

CONFLICT OF INTEREST

The authors declared no competing interests for this work.

FUNDING

This work was supported by JSPS KAKENHI Grant-in-Aid for Scientific Research (C) (grant number 21K06663) from the Japan Society for the Promotion of Science, Takeda Science Foundation, and Mochida Memorial Foundation for Medical and Pharmaceutical Research.

# **Note S1. Data preparation and processing**

In this section, we will outline the detailed data preparation and processing methods. The processed data can be accessed on our Github repository (https://github.com/mizuno-group/GLDADec).

## Blood-derived benchmarking data

The PBMC microarray, PBMC/PMN RNA-Seq, and whole blood RNA-Seq datasets were obtained from the NCBI Gene Expression Omnibus (GEO), with accessions GSE65133, GSE107572, and GSE60424 respectively as processed series matrix files [1–4]. These blood-derived samples are commonly utilized to evaluate deconvolution methods [1–3,5–7]. In addition, we obtained PBMC RNA-Seq data with known more detailed cell type classification, SDY67 and GSE107011 [4,8]. For the SDY67 dataset, we obtained it from Immport database [8]. These datasets were available in the study by Swapna et al [9]. All these datasets were derived from human samples, and the transcript IDs were converted to HGNC symbols using files available from Biomart [10]. Note that only immune cell types measured by flow cytometry and included in the benchmark data were selected for evaluation.

A list of typical marker genes for the cells measured in the benchmark datasets was established using domain knowledge and is provided in Supplementary File S1.

## Brain dataset for benchmarking

We acquired bulk data for the prefrontal cortex from the Religious Orders Study / Memory and Aging Project (ROSMAP) for brain tissue analysis [11]. This dataset offers cell type proportions determined through immunohistochemistry, serving as the ground truth. The processed gene expression matrix were obtained from GitHub repository (https://github.com/ellispatrick/CortexCellDeconv) reported in the original study, and the ground-truth proportions are reported by Swapna et al [9]. Samples that retain ground-truth information ware selected to evaluate estimation performance.

Marker genes for each cell types were defined based on domain knowledge. Supplementary File S2 contains a list of these marker genes.

## Tissue data with perturbation

To assess the estimation of immune cell trafficking in tissues, we retrieved bulk RNA-Seq data from mice and rats with accessions GSE237801 and GSE239996. These data encompass the tissue transcriptome from drug-induced liver injury models and the principal immune cell proportions in the tissues.

Transcripts per kilobase million (TPM) values were obtained for the data, and the transcript IDs were converted to gene symbols. The median values were selected for genes with duplicated names. The control samples and the samples of interest, such as the acetaminophen-treated group, were merged and used as targets to estimate the cellular changes associated with the perturbation. To highlight the differences in the trafficking of immune cells due to perturbation, a feature-wise minmax correction was performed as a preprocessing step to emphasize differences in expression levels between samples.

Cell types verified by flow cytometry were evaluated for deconvolution, including neutrophils, monocytes, NK cells, and Kupffer cells in mice, and B cells, CD4+ T cells, CD8 + T cells, neutrophils, monocytes, and NK cells in rats.

Marker genes for cell types linked to mouse liver were obtained from CellMarker [12]. Cleansing was performed by integrating homologous cell names and eliminating abstract cell names, and finally marker genes specific to the 34 cell types were defined (Supplementary File S3). As there is no established database for marker gene names in rats, we obtained classical cell-type markers that are highly expressed in rats, mice, and humans, as reported in the study by Natasha et al [13].

## Clinical data

To assess the utility of GLDADec in the context of human clinical data, we obtained three distinct types of tumor datasets, comprising 1052 breast invasive carcinoma (BRCA) samples, 577 lung adenocarcinoma (LUAD) samples, and 408 liver hepatocellular carcinoma (LIHC) samples, from the GDC data portal on November 16, 2022 [14]. Marker genes for the cell types associated with each human tissue were obtained from the CellMarker database [12]. The expression were normalized using TPM, and quantile normalization was performed to mitigate the impact of the distribution of expression levels in each sample on the estimated values. Due to the large number of samples and the high computational demands of these datasets, the linear scale expression was transformed into a sparser matrix by dividing it by a constant value of 1000.

## Pseudo lung bulk dataset

The single-cell transcriptome data for cells isolated from human lung tissue was sourced from the Tissue Stability Cell Atlas (TSCA) [15]. A pseudo-bulk dataset was generated comprising five immune cell types (CD4+ T cells, cytotoxic T cells, mast cells, monocytes, and NK cells) and four lung background cell types (alveolar type I, alveolar type II, ciliated cells, and fibroblasts). The generation process involved the following steps: (1) Determining 500 cells each for immune and background types, totaling 1000 cells. (2) Randomly assigning proportions to the constituent cell types under a sum-to-one constraint. (3) Partitioning the data into main and test sets, extracting the corresponding cell counts from the main data, and summing the count data. (4) Repeating this process to create a pseudo-bulk dataset with a chosen sample size. We computed the mean expression level of cells within the test group and established the reference using the same batch as the pseudo-bulk dataset. Additionally, we established a reference derived from a different batch, using accession GSE131907, an independent lung single-cell RNA-Seq dataset published by Kim et al [16].

For TSCA dataset, the raw data was downloaded in h5ad format and processed using the python scanpy library. Ensemble transcript IDs were converted to HGNC symbols using files available from Biomart [10]. For the other dataset, GSE131907, we downloaded raw matrix with Unique molecular identifier and annotation file, respectively. Since this is a txt formant with a huge number of lines, we read each line (barcode) and extracted the cell types of interest for downstream task. This analysis flow is also available on our GitHub repository (https://github.com/mizuno-group/GLDADec).

# **Note S2. Condition of experiments**

In this section, we provide a comprehensive description of the execution environment of the proposed and existing methods in various scenarios, which can be replicated in our GitHub repository (<https://github.com/mizuno-group/GLDADec>).

## Implementation and data collection of other deconvolution methods

This subsection describes the common setting to perform all the benchmarking, including implementation and hyperparameters.

FARDEEP, EPIC, CIBERSORT, DCQ, NNLS, RLR, and Elastic Net are bulk reference deconvolution methods [1,7,17,18]. For FARDEEP, we used the corresponding R package (<https://cran.r-project.org/web/packages/FARDEEP/index.html>) with the default parameter settings to obtain the estimated values of absolute abundance of cells. For EPIC, we employed the online portal (<https://epic.gfellerlab.org/>) to get cell fractions following the default settings. The CIBERSORT website has now been incorporated into the CIBERSORTx website, and we utilized its online portal (<https://cibersortx.stanford.edu/>). All parameters, including the disabling of quantile normalization, were kept at default values. For DCQ, we used the R package (<https://cran.r-project.org/web/packages/ADAPTS/index.html>) implemented by Samuel et al. with the default parameter settings [19]. For the remaining NNLS, RLR, and Elastic Net, we employed the code from the GitHub repository (<https://github.com/mizuno-group/LiverDeconv>) implemented in our previous study with default parameter settings.

For more detailed cell type estimation benchmarking tasks, five state-of-the-art methods leveraging single-cell RNA-Seq or marker gene information were used, including GTM-decon, BayesPrisim, CIBERSORTx, MuSiC, and BSEQ-sc [9,20–23]. GTM-decon offeres three different gene selection methods. We obtained results with the *hvg* method, which selects highly variable genes, a scenario like our method. Additionally, it is known that GTM-decon achieves better performance using multiple topics per cell type, and the reported results were obtained under conditions with five topics per cell type. For BaysPrism, the metadata column for tumor status set to 0 for all cell types, and the online web portal (<https://www.bayesprism.org/>) was utilized. For CIBERSORTx, the settings were identical to those used for CIBERSORT as described above. For the remaining methods, MuSiC and BSEQ-sc, the reported results were obtained using recommended or default settings. Note that the baseline scores for these five state-of-the-art methods were reported in a recent study by Swapna et al. (<https://github.com/li-lab-mcgill/gtm-decon>), and some parameter settings were confirmed by contacting the authors.

## Evaluation metrics for deconvolution

We evaluated the performance using several metrics, including Pearson correlation, rooted mean square error (RMSE), and mean square error (MSE). As a preprocessing step, we adjusted the sum of the proportions of cell types to 1 for both the deconvolution output and the ground-truth. Note that the cell types subject to adjustment are those common between the output and the ground-truth data. In other words, the cell type range constrained by sum to 1 was identical, ensuring comparable evaluations.

## Benchmarking with human blood samples

We employed a linear scaled blood-derived benchmark dataset, utilizing marker gene names defined by domain knowledge (see Supplementary File S1) as partial prior information. Genes exhibiting outliers greater than 2σ from the log-normal distribution of gene expression levels were eliminated for each sample, and 100 genes displaying substantial sample-wide coefficients of variation (CV) were incorporated into the analysis. This number of genes to be added serves as a hyperparameter and is further discussed in the main text. It is important to note that the benchmark data originate from blood samples, and no additional topics were integrated into the model since the constituent cell types are clearly delineated. To correct for the expression level on a linear scale, the values were divided by constants specific to each dataset, i.e., 10, 200, 100, 1000, and 10 for GSE65133, GSE107572, GSE60424, SDY67, and GSE107011, respectively. This operation was implemented to save computational resources and does not alter the relative gene expression levels of each sample.

We conducted a comparison of the performance of GLDADec with seven alternative deconvolution methods, including FARDEEP, EPIC, CIBERSORT, DCQ, NNLS, RLR, and Elastic Net [1,7,17,18]. These methods are all reference-based and require prior knowledge of a gene signature matrix as the reference. The LM22 definition provided by Newman et al. is commonly used as a reference for human blood-derived samples and was also obtained from their work and utilized in our analysis [1]. Notably, LM22 was defined in the same study that generated GSE65133, one of the benchmark datasets used in our analysis, making it a compatible reference. The competing methods have diverse development backgrounds and accept different formats of expression data as input. We adhered to their default settings whenever possible. Specifically, we employed log-normalized gene expression matrices for DCQ and FARDEEP, and a non-log linear scaled matrix for EPIC and CIBERSORT. The remaining NNLS, RLR, and Elastic Net did not specify the input format, so we adapted them to match the scale of the reference data. To eliminate the influence of differences in scale between the analysis object and the reference on the results, we converted LM22 to a log-scale in the DCQ and FARDEEP analysis.

We next compared GLDADec with five state-of-the-art methods, including GTM-decon, BayesPrisim, CIBERSORTx, MuSiC, and BSEQ-sc [9,20–23]. Baseline scores for these methods are reported in recent study by Swapna et al [9]. We downloaded the estimated values for each method and the ground-truth information of cell type proportion from “fig2_data.tar.gz” file stored in GTM-decon's GitHub repository (<https://github.com/li-lab-mcgill/gtm-decon>). In the original benchmark, dendritic cells (DC) and plasmacytoid dendritic cells (pDC) were distinguished, but in this study, pDCs were assumed to be included in DC, and these two cell estimates were combined.

## Comprehensive cell type analysis for mouse perturbed tissue data

The dataset GSE237801 comprises bulk RNA-Seq data from mouse liver tissue under various compound administration and immune cell proportions measured by flow cytometry, which is a suitable benchmark dataset for deconvolution methods for tissues [24]. This study primarily focused on the acetaminophen (APAP) and alpha-naphthyl isothiocyanate (ANIT) treatment groups, examining the performance of deconvolution methods in detecting perturbation-induced changes in immune cell proportions relative to control samples. It is important to note that the tissue sample is a heterogeneous cell population, and subsequent analyses incorporate additional modeling considerations.

In our analysis of benchmarking using human blood datasets, GLDADec utilized linear scale expression levels as input and marker genes as prior information. In the examination of tissues, it is anticipated that transcriptome variation will arise due to the presence of unknown cells and confounding factors. To address this, we gathered liver-related cell types and their associated marker genes in a data-driven manner from the CellMarker database [12]. Moreover, to account for the diverse factors affecting the tissue transcriptome, we included 1000 genes with substantial coefficients of variation, which adequately describe the state of the tissue.

We contrasted the performance of GLDADec with seven existing reference-based methods, which are identical to those analyzed using the blood-derived benchmark dataset. Since the LM22 expression levels are derived from human samples, we employed the mouse-derived LM13, as previously defined in our analysis, as a reference to account for species differences in the mouse data. Additionally, we performed an analysis in which differentially expressed gene (DEGs) names derived from the same LM13 were considered as markers and subjected to GLDADec.

## Application to rat data

GSE239996 contains data on rat liver tissue bulk RNA-Seq under various compound administration and immune cell proportions measured by flow cytometry [25]. In this study, we concentrated on the acetaminophen (APAP) treatment groups and assessed the efficacy of the deconvolution method in detecting immune cell proportion alterations induced by the compound compared to control samples. Similar to the analysis of the mouse data, 1000 genes with significant expression variation were included.

In this section, we defined three types of marker gene names for GLDADec analysis. The first set of classical cell type markers were collected from Figure 4A of Natasha's work [13]. The second marker is the same as defined in the comprehensive analysis of mice from CellMarker. For the last set, DEGs were calculated for the six cell types (LM6) out of LM13, and the corresponding gene names were designated as markers. Utilizing LM6 as a reference, we compared its performance with seven competing methods.

## Application to tumor samples

The TCGA RNA-seq data were obtained from numerous experiments and consist of a large number of samples, but also various confounding factors. To address this, we performed quantile normalization and aligned the distribution of each sample. However, the policy of eliminating genes with values that are more than two standard deviations away from the mean did not ensure enough genes, so we instead included genes with large expression variation in this analysis.

We gathered marker gene names from the background tissues of each cancer subtype using CellMarker and utilized this information as prior knowledge. Specifically, we obtained markers for 47, 73, and 80 cell types in a data-driven manner for breast, lung, and liver cancers, respectively. It is important to note that these markers were derived from human clinical data. Marker genes in human tissues have been well studied and are informative and comprehensive. Therefore, we performed modeling considering only one additional topic as “others”.

## Application to pseudo lung bulk dataset

We included 100 genes with large coefficients of variation in the analysis, without considering additional topics. The setting is the same as for the analysis of blood samples, and this is since the cell types that comprise the sample are relatively explicit. In addition, the count data was corrected by dividing the count data by a constant of 100 to reduce the computational cost when GLDADec was performed.

# **Note S3. Proof of 1.5**

A Dirichlet distribution is defined with parameter $\boldsymbol{\alpha}=\left( \alpha_{1}, \alpha_{2}, \ldots, \alpha_{K} \right)$ as follows:

$$p\left( \boldsymbol{\pi} \right| \boldsymbol{\alpha})=Dir\left( \boldsymbol{\pi} \right| \boldsymbol{\alpha}) \equiv\frac{\Gamma\left( \sum_{k=1}^{K} \alpha_{k} \right)}{\prod_{k=1}^{K} \Gamma\left( \alpha_{k} \right)}\prod_{k=1}^{K} \pi_{k}^{\boldsymbol{\alpha}_{k}-1},$$

where, considering the graphical model of Latent Dirichlet Allocation (LDA), we obtain the following equation:

$$p\left( \boldsymbol{G},\boldsymbol{Z},\boldsymbol{\theta}, \boldsymbol{\varphi} | \boldsymbol{\alpha},\boldsymbol{\eta} \right)=\left( \prod_{k} p\left( \boldsymbol{\varphi}_{k} | \boldsymbol{\eta} \right) \right)\left( \prod_{m} p\left( \boldsymbol{\theta}_{m} | \boldsymbol{\alpha} \right)\prod_{n} p\left( Z_{m,n} | \boldsymbol{\theta}_{m} \right)p\left( G_{m,n} | \boldsymbol{\varphi}_{k}, Z_{m,n} \right) \right).$$

Now, to consider the joint distribution of $p\left( \boldsymbol{G,Z} \right| \boldsymbol{\alpha}, \boldsymbol{\eta})$, both $\boldsymbol{\theta}$ and $\boldsymbol{\varphi}$ were integrated out. Let $n_{k,v}= \sum_{m=1}^{M} \sum_{i=1}^{n_{m}} \delta(z_{m,n}=k, G_{m,n}=v)$ and $n_{m,k}= \sum_{i=1}^{n_{m}} \delta(z_{m,n}=k)$, and the equation is written as follows:

$$p\left( \boldsymbol{G,Z} \right| \boldsymbol{\alpha}, \boldsymbol{\eta})= \int p\left( \boldsymbol{G},\boldsymbol{Z},\boldsymbol{\theta}, \boldsymbol{\varphi} | \boldsymbol{\alpha},\boldsymbol{\eta} \right)d\boldsymbol{\theta}d\boldsymbol{\varphi}$$

$$= \int\left[ \prod_{n} p\left( Z_{m,i} | \boldsymbol{\theta}_{m} \right)p\left( G_{m,n} | \boldsymbol{\varphi}_{k}, Z_{m,n} \right) \right]\cdot\left[ \prod_{k} p\left( \boldsymbol{\varphi}_{k} | \boldsymbol{\eta} \right) \right]\cdot\left[ \prod_{m} p\left( \boldsymbol{\theta}_{m} | \boldsymbol{\alpha} \right) \right]d\boldsymbol{\theta}d\boldsymbol{\varphi}$$

$$= \int\prod_{k=1}^{K} \left[ \prod_{v=1}^{V} \varphi_{k,v}^{n_{k,v}} \right]p\left( \boldsymbol{\varphi}_{k} | \boldsymbol{\eta} \right)d\boldsymbol{\varphi}_{k} \cdot\int\prod_{m=1}^{M} \left[ \prod_{k=1}^{K} \theta_{m, k}^{n_{m,k}} \right]p\left( \boldsymbol{\theta}_{m} | \boldsymbol{\alpha} \right)d\boldsymbol{\theta}_{m}$$

$$= \int\prod_{k=1}^{K} \left[ \prod_{v=1}^{V} \varphi_{k,v}^{n_{k,v}} \right]\frac{\Gamma\left( \sum_{v=1}^{V} \eta_{v} \right)}{\prod_{v=1}^{V} \Gamma\left( \eta_{v} \right)} \prod_{v=1}^{V} \varphi_{k,v}^{\eta_{v}-1}d\boldsymbol{\varphi}_{k} \cdot\int\prod_{m=1}^{M} \left[ \prod_{k=1}^{K} \theta_{m, k}^{n_{m,k}} \right]\frac{\Gamma\left( \sum_{k=1}^{K} \alpha_{k} \right)}{\prod_{k=1}^{K} \Gamma\left( \alpha_{k} \right)} \prod_{k=1}^{K} \theta_{m,k}^{\alpha_{k}-1}d\boldsymbol{\theta}_{m}$$

$$=\int\prod_{k=1}^{K} \frac{\Gamma\left( \sum_{v=1}^{V} \eta_{v} \right)}{\prod_{v=1}^{V} \Gamma\left( \eta_{v} \right)} \left[ \prod_{v=1}^{V} \varphi_{k,v}^{n_{k,v}+\eta_{v}-1} \right]d\boldsymbol{\varphi}_{k} \cdot\int\prod_{m=1}^{M} \frac{\Gamma\left( \sum_{k=1}^{K} \alpha_{k} \right)}{\prod_{k=1}^{K} \Gamma\left( \alpha_{k} \right)} \left[ \prod_{k=1}^{K} \theta_{m, k}^{n_{m,k}+\alpha_{k}-1} \right]d\boldsymbol{\theta}_{m}$$

$$=\prod_{k=1}^{K} \frac{\Gamma\left( \sum_{v=1}^{V} \eta_{v} \right)}{\prod_{v=1}^{V} \Gamma\left( \eta_{v} \right)}\frac{\prod_{v=1}^{V} \Gamma\left( n_{k,v}+ \eta_{v} \right)}{\Gamma\left( \sum_{v} n_{k,v}+ \eta_{v} \right)}\int p\left( \boldsymbol{\varphi}_{k} | \boldsymbol{G},\boldsymbol{Z},\boldsymbol{\eta} \right)d\boldsymbol{\varphi}_{k}\cdot\prod_{m=1}^{M} \frac{\Gamma\left( \sum_{k=1}^{K} \alpha_{k} \right)}{\prod_{k=1}^{K} \Gamma\left( \alpha_{k} \right)}\frac{\prod_{k=1}^{K} \Gamma\left( n_{m,k}+ \alpha_{k} \right)}{\Gamma\left( \sum_{k} n_{m,k}+ \alpha_{k} \right)}\int p\left( \boldsymbol{\theta}_{m} | \boldsymbol{Z}_{m},\boldsymbol{\alpha} \right)d\boldsymbol{\theta}_{m}$$

$$\begin{aligned} \prod_{k=1}^{K} \frac{\Gamma\left( \sum_{v=1}^{V} \eta_{v} \right)}{\prod_{v=1}^{V} \Gamma\left( \eta_{v} \right)}\frac{\prod_{v=1}^{V} \Gamma\left( n_{k,v}+ \eta_{v} \right)}{\Gamma\left( \sum_{v} n_{k,v}+ \eta_{v} \right)}\cdot\prod_{m=1}^{M} \frac{\Gamma\left( \sum_{k=1}^{K} \alpha_{k} \right)}{\prod_{k=1}^{K} \Gamma\left( \alpha_{k} \right)}\frac{\prod_{k=1}^{K} \Gamma\left( n_{m,k}+ \alpha_{k} \right)}{\Gamma\left( \sum_{k} n_{m,k}+ \alpha_{k} \right)}. \#\left( 1.5 \right) \end{aligned}$$

**References**

1. Newman AM, Liu CL, Green MR, et al. Robust enumeration of cell subsets from tissue expression profiles. Nat Methods 2015; 12:453–457

2. Finotello F, Mayer C, Plattner C, et al. Molecular and pharmacological modulators of the tumor immune contexture revealed by deconvolution of RNA-seq data. Genome Med 2019; 11:34

3. Linsley PS, Speake C, Whalen E, et al. Copy Number Loss of the Interferon Gene Cluster in Melanomas Is Linked to Reduced T Cell Infiltrate and Poor Patient Prognosis. PLoS One 2014; 9:e109760

4. Monaco G, Lee B, Xu W, et al. RNA-Seq Signatures Normalized by mRNA Abundance Allow Absolute Deconvolution of Human Immune Cell Types. Cell Rep 2019; 26:1627-1640.e7

5. Menden K, Marouf M, Oller S, et al. Deep learning–based cell composition analysis from tissue expression profiles. Sci Adv 2020; 6:

6. Andrade Barbosa B, van Asten SD, Oh JW, et al. Bayesian log-normal deconvolution for enhanced in silico microdissection of bulk gene expression data. Nat Commun 2021; 12:6106

7. Racle J, de Jonge K, Baumgaertner P, et al. Simultaneous enumeration of cancer and immune cell types from bulk tumor gene expression data. Elife 2017; 6:

8. Haralambieva IH, Painter SD, Kennedy RB, et al. The Impact of Immunosenescence on Humoral Immune Response Variation after Influenza A/H1N1 Vaccination in Older Subjects. PLoS One 2015; 10:e0122282

9. Swapna LS, Huang M, Li Y. GTM-decon: guided-topic modeling of single-cell transcriptomes enables sub-cell-type and disease-subtype deconvolution of bulk transcriptomes. Genome Biol 2023; 24:190

10. Smedley D, Haider S, Ballester B, et al. BioMart – biological queries made easy. BMC Genomics 2009; 10:22

11. Patrick E, Taga M, Ergun A, et al. Deconvolving the contributions of cell-type heterogeneity on cortical gene expression. PLoS Comput Biol 2020; 16:e1008120

12. Hu C, Li T, Xu Y, et al. CellMarker 2.0: an updated database of manually curated cell markers in human/mouse and web tools based on scRNA-seq data. Nucleic Acids Res 2023; 51:D870–D876

13. Qaisar N, Arowosegbe A, Derr AG, et al. Type I IFN–Driven Immune Cell Dysregulation in Rat Autoimmune Diabetes. Immunohorizons 2021; 5:855–869

14. Grossman RL, Heath AP, Ferretti V, et al. Toward a Shared Vision for Cancer Genomic Data. New England Journal of Medicine 2016; 375:1109–1112

15. Madissoon E, Wilbrey-Clark A, Miragaia RJ, et al. scRNA-seq assessment of the human lung, spleen, and esophagus tissue stability after cold preservation. Genome Biol 2020; 21:1

16. Kim N, Kim HK, Lee K, et al. Single-cell RNA sequencing demonstrates the molecular and cellular reprogramming of metastatic lung adenocarcinoma. Nat Commun 2020; 11:2285

17. Hao Y, Yan M, Heath BR, et al. Fast and robust deconvolution of tumor infiltrating lymphocyte from expression profiles using least trimmed squares. PLoS Comput Biol 2019; 15:e1006976

18. Altboum Z, Steuerman Y, David E, et al. Digital cell quantification identifies global immune cell dynamics during influenza infection. Mol Syst Biol 2014; 10:

19. Danziger SA, Gibbs DL, Shmulevich I, et al. ADAPTS: Automated deconvolution augmentation of profiles for tissue specific cells. PLoS One 2019; 14:e0224693

20. Chu T, Wang Z, Pe’er D, et al. Cell type and gene expression deconvolution with BayesPrism enables Bayesian integrative analysis across bulk and single-cell RNA sequencing in oncology. Nat Cancer 2022; 3:505–517

21. Steen CB, Liu CL, Alizadeh AA, et al. Profiling Cell Type Abundance and Expression in Bulk Tissues with CIBERSORTx. Methods Mol Biol 2020; 2117:135–157

22. Wang X, Park J, Susztak K, et al. Bulk tissue cell type deconvolution with multi-subject single-cell expression reference. Nat Commun 2019; 10:380

23. Baron M, Veres A, Wolock SL, et al. A Single-Cell Transcriptomic Map of the Human and Mouse Pancreas Reveals Inter- and Intra-cell Population Structure. Cell Syst 2016; 3:346-360.e4

24. Azuma I, Mizuno T, Morita K, et al. Investigation of the usefulness of liver-specific deconvolution method by establishing a liver benchmark dataset. NAR Genom Bioinform 2024; 6:

25. Morita K, Mizuno T, Azuma I, et al. Rat deconvolution as knowledge miner for immune cell trafficking from toxicogenomics databases. Toxicological Sciences 2023;
